# Virus‐Induced Histone Lactylation Promotes Virus Infection in Crustacean

**DOI:** 10.1002/advs.202401017

**Published:** 2024-06-14

**Authors:** Yu Zhang, Xiaobo Zhang

**Affiliations:** ^1^ College of Life Sciences Zhejiang University Hangzhou 310058 P. R. China; ^2^ Department of Clinical Pharmacology Key Laboratory of Clinical Cancer Pharmacology and Toxicology Research of Zhejiang Province Affiliated Hangzhou First People's Hospital Cancer Center Westlake University School of Medicine Hangzhou 310006 P. R. China; ^3^ Laboratory for Marine Biology and Biotechnology of Pilot National Laboratory for Marine Science and Technology (Qingdao) Qingdao 266003 P. R. China; ^4^ Southern Marine Science and Engineering Guangdong Laboratory (Zhuhai) Zhuhai 519000 P. R. China

**Keywords:** glycolysis, histone lactylation, metabolite, miRNA, virus infection

## Abstract

As “non‐cellular organisms”, viruses need to infect living cells to survive themselves. The virus infection must alter host's metabolisms. However, the influence of the metabolites from the altered metabolisms of virus‐infected host cells on virus‐host interactions remains largely unclear. To address this issue, shrimp, a representative species of crustaceans, is challenged with white spot syndrome virus (WSSV) in this study. The in vivo results presented that the WSSV infection enhanced shrimp glycolysis, leading to the accumulation of lactate. The lactate accumulation in turn promoted the site‐specific histone lactylation (H3K18la and H4K12la) in a p300/HDAC1/HDAC3‐dependent manner. H3K18la and H4K12la are enriched in the promoters of 75 target genes, of which the H3K18la and H4K12la modification upregulated the expression of ribosomal protein S6 kinases 2 (*S6K2*) in the virus‐infected hosts to promote the virus infection. Further data revealed that the virus‐encoded miR‐N20 targeted hypoxia inducible factor‐1α (*HIF‐1α*) to inhibit the host glycolysis, leading to the suppression of H3K18la and H4K12la. Therefore, the findings contributed novel insights into the effects and the underlying mechanism of the virus‐induced histone lactylation on the virus‐host interactions, providing new targets for the control of virus infection.

## Graphical abstract

Virus infection alters the metabolism of host cells. However, the role of the virus‐induced metabolites in the virus‐host interactions is largely unknown. In this study, the results show that the virus infection enhances glycolysis of crustacean to generate lactate, leading to the site‐specific histone lactylation (H3K18la and H4K12la). The histone lactylation promotes the virus infection via target genes.

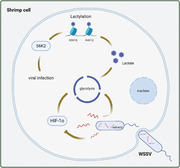

## 1 Introduction

Over the past decades, we have witnessed a significant number of high‐profile outbreaks of new and re‐emerging viruses, such as severe acute respiratory syndrome coronavirus 2 (SARS‐CoV‐2), avian influenza virus, Middle East respiratory syndrome coronavirus, Zika virus, and Ebola virus.^[^ ^1^ ^]^ Viruses are one of the greatest threats to human health, thus attracting worldwide attention. When viruses infect host cells, they almost invariably cause metabolic changes in the infected cells.^[^ ^2^ ^]^ The virus itself is metabolically inert and must rely on the metabolic machines of host cells to produce its components and replicate new copies of the virus.^[^ ^3^ ^]^ There are multiple mechanisms by which a virus infection can induce metabolic changes in infected cells. A common consequence of the virus infection is the induction of high glucose metabolism, causing aerobic glycolysis (the so‐called Warburg effect) in cells and altering the nature of lipid metabolism, usually from fatty acid oxidation to fatty acid synthesis.^[^ ^4^ ^]^ As reported, during the infection of white spot syndrome virus (WSSV) into shrimp, the compound palmitamide encapsulated in the viral particles enhances the glycolytic process to promote the virus infection by binding to the glycolytic rate‐limiting enzyme, triosephosphate isomerase (TPI), to enhance the activity of TPI.^[^ ^5^ ^]^ The alterations of host metabolisms resulted from the virus infection are involved in or/and lead to the dysregulation of gene expressions and the modifications of proteins associated with host metabolisms.

In host cells, the virus infection can induce a series of reversible epigenetic modifications that regulate gene expressions, such as histone methylation and acetylation, DNA/RNA methylation, chromatin remodeling, and non‐coding RNAs.^[^ ^6^ ^]^ It is found that the infection of hepatitis B virus (HBV) activates histone acetyltransferase 1 to promote the acetylation of histone H3K27/H4K5/H4K12 on cccDNA minichromosome by HBx‐co‐activated transcriptional factor specificity protein 1 in a positive feedback manner.^[^ ^7^ ^]^ HBV can not only change histone acetylation of host cells, but also directly affect histone methylation modification. HBV X protein (HBx) plays a crucial role in this process.^[^ ^8^ ^]^ HBx, in acute liver failure, suppresses solute carrier family 7 member 11 expression through H3K27me3 modification by enhancer of zeste homolog 2.^[^ ^8^ ^]^ In hepatoma cells, HBx directly recruits DNA methyltransferase 3A to the promoter region of the NAD(P)H:quinone oxidoreductase 1 (*NQO1*) gene to induce epigenetic silencing of *NQO1*.^[^ ^9^ ^]^ In influenza A virus (IAV), the non‐structural protein 1 of IAV is found to directly bind to the cellular DNA methyltransferase 3B, thereby inhibiting the methylation of the promoters of genes encoding suppressors of JAK‐STAT signaling pathway.^[^ ^10^ ^]^ Viruses can also regulate epigenetic inheritance through virus‐encoded microRNAs (miRNAs).^[^ ^11^ ^]^ In heliothiszea nudivirus‐1 (HzNV‐1)‐infected insect cells, the virus‐encoded miR‐420 suppresses the expression of histone modification‐associated enzyme su(var)3‐9 to promote HzNV‐1 infection by histone methylation.^[^ ^11^ ^]^ SARS‐CoV‐2 induces metabolic suppression of oxidative phosphorylation and the tricarboxylic acid cycle in multiple organs and epigenetic changes of DNA methylation, which can, in part, contribute to COVID‐19 pathogenesis.^[^ ^12^ ^]^ The virus infection enhances glycolysis and the production of lactate to promote virus invasion.^[^ ^5^ ^]^ Glycolysis can regulate histone lactylation by affecting intracellular lactate levels, playing a significant role in various diseases and biological processes.^[^ ^13^ ^]^ At present, however, the influence of the metabolites generated from the altered metabolisms of the virus‐infected host cells on host immunity and virus infection has not been extensively investigated.

To explore the underlying mechanisms of the virus‐induced metabolites in host antiviral immunity, a crustacean shrimp were challenged with WSSV and then the host metabolites were characterized. The results indicated that the virus infection enhanced the host glycolysis and the glycolysis‐derived lactate promoted histone lactylation (H3K18la and H4K12la) to facilitate the virus infection.

## 2 Results

### 2.1 Promotive Impact of Virus Infection on Histone Lactylation

To explore the influence of the virus infection on host glycolysis, shrimp were challenged with WSSV and then the content of lactate in shrimp hemocytes and intestinal tissues was examined. The results showed that the relative content of lactate in the hemocytes and intestinal tissues of WSSV‐infected shrimp was significantly increased compared to the PBS control (Figure S1A, Supporting Information), suggesting that glycolysis was enhanced in shrimp in response to virus challenge.

To further confirm the involvement of glycolysis in the virus infection, shrimp were infected with WSSV, followed by examining the expression levels of hexokinase (HK) and lactate dehydrogenase (LDH), the key enzymes of glycolysis. Quantitative real‐time PCR results showed that the expressions of *HK* and *LDH* significantly increased in the hemocytes and intestinal tissues of WSSV‐infected shrimp (Figure S1B, Supporting Information). At the same time, the enzymatic activities of HK and LDH were significantly increased in the hemocytes of WSSV‐infected shrimp compared to the PBS control (Figure S1C, Supporting Information). These data indicated that the virus infection promoted host's glycolysis.

The acidification of cells can promote internalization and phagocytosis,^[^ ^18^ ^]^ while the enhancement of glycolysis can generate numerous lactate. Therefore, the impact of WSSV infection on internalization of shrimp hemocytes was further evaluated. The results showed that the WSSV infection significantly increased the internalization percentage of shrimp hemocytes compared to the control (PBS) (Figure S1D, Supporting Information), suggesting that the enhancement of the virus‐mediated glycolysis promoted host's internalization.

These findings presented that the virus infection enhanced host glycolysis, leading to the accumulation of lactate, thus further promoting host's internalization.

**Figure 1:**
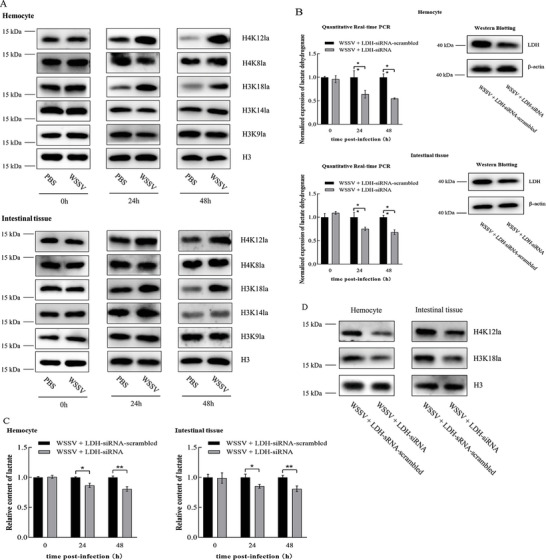
Promotive impact of virus infection on histone lactylation. A) Influence of virus infection on the histone lactylation. Shrimp were challenged with WSSV. At different time post‐infection, the hemocytes and intestinal tissues of shrimp were subjected to the detection of the histone lactylation using Western blot. the specific antibody against the lactylation sites of H3K9, H3K14, H3K18, H4K8 or H4K12 was used. H3 was used as a control. B) Silencing of lactate dehydrogenase (LDH) in shrimp. Shrimp were simultaneously injected with WSSV and LDH‐siRNA or LDH‐siRNA‐scrambled. At different time after injection, the expression level of LDH in the hemocytes and intestinal tissues of shrimp was examined using quantitative real‐time PCR (*p < 0.05; **p < 0.01). C) Impact of LDH silencing on glycolysis of WSSV‐infected shrimp. The lactate content in the hemocytes and intestinal tissues of shrimp injected with WSSV and LDH‐siRNA or siRNA‐scrambled was evaluated (*p < 0.05; **p < 0.01). As a control, siRNA‐scrambled was included in the injection. D) Influence of LDH silencing on the histone lactylation in the WSSV‐infected shrimp. Shrimp were injected with WSSV and LDH‐siRNA or siRNA‐scrambled. Forty‐eight hours later, the hemocytes and intestinal tissues of shrimp were subjected to Western blot to examine the lactylation of histone H3K18 and H4K12. H3 was used as a control. E) Impact of 2‐deoxy‐D‐glucose on the content of lactate in WSSV‐infected shrimp. The shrimp were injected with WSSV and 2‐deoxy‐D‐glucose or PBS. At different time post‐infection, the contents of lactate in the hemocytes and intestinal tissue of shrimp were examined (**p < 0.01). F) Western blot analysis of lactylation of H3K18 and H4K12 in shrimp. The hemocytes and intestinal tissues of shrimp treated with WSSV, and 2‐deoxy‐D‐glucose or PBS were collected at 48 h post‐infection and then subjected to Western blot to detect the lactylation levels of H3K18 and H4K12. H3 was used as a control. G) Impact of sodium lactate (Nala) on the lactate content of WSSV‐infected shrimp. Shrimp were simultaneously injected with WSSV and Nala or PBS. At different time points post‐infection, the lactate contents in the hemocyte and intestinal tissue of shrimp were examined (**p < 0.01). H) Western blotting analysis of the lactylation of H3K18 and H4K12 in shrimp treated with WSSV and Nala. Shrimp were injected with WSSV and Nala. At 48 h post‐infection, the hemocytes and intestinal tissues were subjected to Western blot to evaluate the levels of H3K18la and H4K12la. H3 was used as a control.

To reveal the underlying mechanism of virus‐induced lactate on the gene expression during virus‐host interactions, the histone lactylation was characterized in the hemocytes and intestinal tissues of shrimp challenged with WSSV. The results showed that the WSSV challenge significantly increased the lactylation level of H3K18 and H4K12 but not H3K9, H3K14 and H4K8 (**Figure** **1**A), indicating that the virus infection promoted the lactylation of H3K18 and H4K12 (H3K18la and H4K12la).

To assess whether the site‐specific histone lactylation induced by the virus infection was mediated by glycolysis, the expression of *LDH* gene was knocked down using LDH‐siRNA in the WSSV‐infected shrimp, followed by the examination of histone lactylation. The results revealed that the expression of LDH was silenced in the hemocytes, and intestinal tissues compared to the siRNA‐scrambled control (Figure 1B). The LDH silencing led to a significant decrease of lactate content in the hemocytes and intestinal tissues of the WSSV‐challenged shrimp (Figure 1C). At the same time, the Western blot data showed that the H3K18la and H4K12la levels were significantly decreased in the hemocytes, and intestinal tissues of shrimp treated with LDH‐siRNA and WSSV compared with the control (Figure 1D). These results indicated that the histone lactylation induced by the virus infection was mediated by glycolysis.

To further confirm the involvement of glycolysis in the histone lactylation induced by the virus infection, the WSSV‐infected shrimp were treated with 2‐deoxy‐D‐glucose (2‐DG), a glucose analogue that acts as a competitive glycolysis inhibitor, and then the histone lactylation in the hemocytes and intestinal tissues was examined. The results showed that the 2‐DG treatment significantly decreased the content of lactate in the WSSV‐infected shrimp (Figure 1E). At the same time, the levels of H3K18la and H4K12la were obviously decreased in the hemocytes, and intestinal tissues of shrimp treated with WSSV and 2‐DG (Figure 1F). These data demonstrated that the virus‐induced histone lactylation was mediated via glycolysis.

To assess whether the histone lactylation was mediated by lactate, sodium lactate (Nala) was injected to the WSSV‐infected shrimp. The result indicated that the lactate content in the hemocytes and intestinal tissues of shrimp treated with Nala was significantly increased (Figure 1G). At the same time, the levels of H3K18la and H4K12la were obviously increased in the hemocytes, and intestinal tissues of shrimp treated with WSSV and Nala (Figure 1H). These results showed that the histone lactylation of WSSV‐challenged shrimp was mediated by lactate.

Taken together, these data demonstrated that the virus infection promoted the site‐specific histone lactylation (H3K18la and H4K12la) via enhancing the host glycolysis.

### 2.2 Mechanism of Site‐Specific Lactylation In Vivo

**Figure 2:**
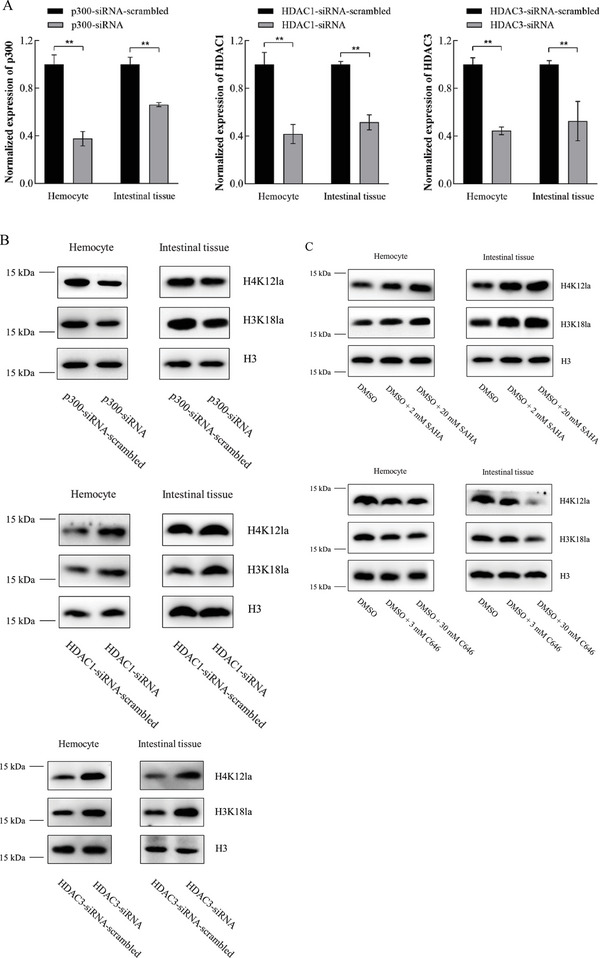
Mechanism of site‐specific lactylation in vivo. A) Silencing of p300, HDAC1 and HDAC3 in shrimp. Shrimp were co‐injected with WSSV and siRNA (p300‐siRNA, HDAC1‐siRNA or HDAC3‐siRNA). At different time after the injection (12, 24, and 36 h), siRNA alone was injected into the same shrimp. As controls, p300‐siRNA‐scramble, HDAC1‐siRNA‐scramble and HDAC3‐siRNA‐ scramble were included in the injections. At 48 h after the first injection, the expression levels of p300, HDAC1 and HDAC3 in the hemocytes and intestinal tissues of shrimp were examined (**p < 0.01). B) Roles of p300, HDAC1 and HDAC3 in the lactylation of H3K18 and H4K12 in shrimp. The expression of p300, HDAC1 or HDAC3 was silenced in the WSSV‐infected shrimp using sequence‐specific siRNA. At 48 h after the first injection of siRNA, the shrimp hemocytes and intestinal tissues were subjected to Western blot analysis to detect the levels of H3K18la and H4K12la. H3 was used as a control. C) Influence of inhibitor of p300 or HDAC1 and HDAC3 on the lactylation of H3K18 and H4K12 in shrimp. The WSSV‐infected shrimp were treated with Vorinostat (SAHA), an inhibitor of histone deacetylase or C646, an inhibitor of p300, at different concentrations. At different time after treatment, the H3K18la and H4K12la levels in shrimp hemocytes and intestinal tissues were examined using Western blot. H3 was used as a control.

To reveal the mechanism of site‐specific histone lactylation, the genes encoding the acetyltransferase p300 responsible for histone lactylation and the deacetylases HDAC1 (histone deacetylase 1) and HDAC3 responsible for histone delactylation were respectively silenced in the WSSV‐infected shrimp using the sequence‐specific siRNA, followed by the examination of H3K18la and H4K12la. The results indicated that the sequence‐specific siRNA significantly decreased the expression of p300, HDAC1 or HDAC3 in shrimp hemocytes and intestinal tissues compared to the control (**Figure** **2**A).

The silencing of p300 led to significant decreases of the H3K18la and H4K12la levels in shrimp hemocytes and intestinal tissues, while the HDAC1 or HDAC3 silencing resulted in increases of the H3K18la and H4K12la levels compared to the controls (Figure 2B). To further confirm the roles of p300, HDAC1 and HDAC3 in the lactylation of H3K18 and H4K12 in shrimp, the WSSV‐infected shrimp were treated with Vorinostat (SAHA), an inhibitor of histone deacetylase (HDAC), or C646, an inhibitor of p300. The sequence alignment showed that the catalytic domains of HDAC1, HDAC3 and p300 were highly conserved in shrimp and human being (Figure S2, Supporting Information). Therefore, SAHA and C646 were used to suppress the activities of HDAC1, HDAC3 or p300 in this study. Western blots showed that SAHA and C646 obviously increased and reduced the H3K18la and H4K12la levels in shrimp hemocytes and intestinal tissues in a concentration‐dependent manner, respectively (Figure 2C). These data indicated that shrimp p300 acted as a “writer” of site‐specific histone lactylation, while shrimp HDAC1 and HDAC3 exerted the roles of histone lysine delactylases.

### 2.3 Target Genes Directly Regulated by H3K18la and H4K12la

**Figure 3:**
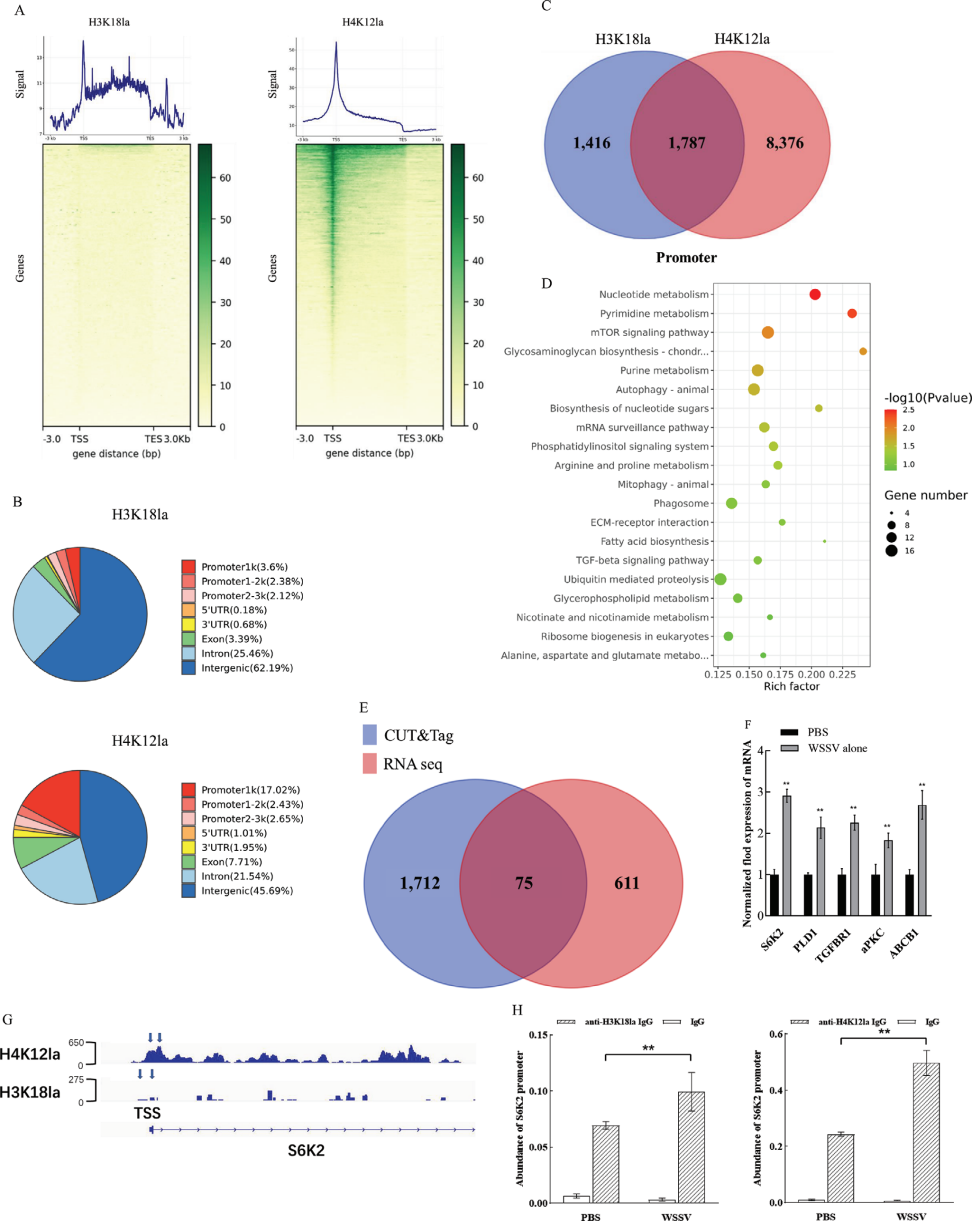
Target genes directly regulated by H3K18la and H4K12la. A) Genome‐wide enrichment analysis of DNAs bound to H3K18la and H4K12la. Shrimp were infected with WSSV. Forty‐eight hours later, the hemocytes of WSSV‐infected shrimp were subjected to the CUT&Tag analysis. The heatmap presented the counts of genes bound to H3K18la (left) or H4K12la (right). The binding density of genes with transcription start site (TSS) and transcription end site (TES) was visualized using deepTools. B) Genome‐wide distribution of the DNAs bound to H3K18la or H4K12la. C) Number of target genes whose promotors were bound to H3K18la and/or H4K12la. D) Kyoto encyclopedia of genes and genomes (KEGG) analysis of the target genes regulated by H3K18la and H4K12la. E) Number of the genes targeted by H3K18la and H4K12la. Shrimp were challenged by WSSV. At 48 h after infection, the shrimp hemocytes were subjected to transcriptome analysis. A total of 75 upregulated genes were overlapped in the CUT&Tag and RNA‐seq data. F) Effects of WSSV infection on the expressions of target genes of H3K18la and H4K12la. Shrimp were injected with WSSV or PBS. PBS was included in the injection as a control. At 48 h after injection, the expression profiles of shrimp ribosomal protein S6 kinases 2 (S6K2), phospholipase D1 (PLD1), TGF‐beta receptor type‐1 (TGFBR1), atypical protein kinase C (aPKC) and ATP‐dependent translocase (ABCB1) in the shrimp hemocytes were examined using quantitative real‐time PCR (**p < 0.01). G) Genome browser tracks of CUT&Tag signals at the *S6K2* gene loci. The blue arrows indicated the peak regions of H3K18la and H4K12la around the transcription start sites (TSS) and the enrichment at the target gene's promoters. The number showed the peak height. H) H3K18la and H4K12la levels in the *S6K2* promoter of WSSV‐infected shrimp. Shrimp were injected with WSSV or PBS. PBS was used as a control. At 48 h after injection, ChIP‐qPCR assay was performed to detect the enrichment of H3K18la and H4K12la in the *S6K2* promoter (**p < 0.01).

To explore the underlying mechanism mediated by the site‐specific histone lactylation (H3K18la or H4K12la), the target genes directly regulated by H3K18la or H4K12la were characterized. The hemocytes of WSSV‐infected shrimp were subjected to the genome‐wide CUT&Tag analysis. The results showed that the binding peaks of H3K18la were mainly distributed in the coding regions (exon and intron) of 11363 target genes, while the binding peaks of H4K12la were mainly enriched in the promoter regions of 14775 target genes (**Figure** **3**A and Supporting information of sequences). Most of the DNAs bound to H3K18la and H4K12la belonged to the intergenic regions. Among them, 8.1% or 22.1% DNAs bound to H3K18la or H4K12la were located within the promoter sequences (Figure 3B). The promoters of 1787 genes were bound to H3K18la and H4K12la (Figure 3C), indicating that the expressions of these target genes were regulated by the site‐specific histone lactylation (H3K18la and H4K12la). Kyoto encyclopedia of genes and genomes (KEGG) analysis revealed that the target genes regulated by H3K18la and H4K12la were enriched in metabolic pathways and cellular immunology‐related pathways (Figure 3D), suggesting the regulatory roles of histone lactylation in virus‐host interactions.

To uncover the regulatory roles of H3K18la and H4K12la in gene expression, the transcriptomes of WSSV‐challenged and non‐treated shrimp hemocytes were performed. The results indicated that 75 potential target genes were significantly upregulated in the WSSV‐infected shrimp with H3K18la and H4K12la (Figure 3E; Figure S3, Supporting Information), showing that these genes were the target genes of H3K18la and H4K12la. To verify these results, 5 of 75 potential target genes, including shrimp ribosomal protein S6 kinases 2 (*S6K2*), phospholipase D1 (*PLD1*), TGF‐beta receptor type‐1 (*TGFBR1*), atypical protein kinase C (*aPKC*) and ATP‐dependent translocase (*ABCB1*), were randomly selected to conduct quantitative real‐time PCR. The data revealed that *S6K2*, *PLD1*, *TGFBR1*, *aPKC* and *ABCB1* were significantly upregulated in the hemocytes of WSSV‐infected shrimp compared to the control (Figure 3F), thus confirming the results of RNA sequencing.

Based on the KEGG data of 75 potential target genes, S6K2, a downstream effector of mTORC1 signaling pathway, was further characterized (Figure S3, Supporting Information). As reported, mTORC1 plays an essential effect on virus infection by regulating autophagy.^[^ ^19^ ^]^ To confirm that the upregulation of *S6K2* was activated by H3K18la and H4K12la in the promoter region, the genomic visualization analysis of the CUT&Tag signals at the *S6K2* promoter was performed. The results showed a significant enrichment of multiple H3K18la and H4K12la signal peaks at the *S6K2* promoter region in the WSSV‐infected shrimp hemocytes (Figure 3G). At the same time, the ChIP‐qPCR analysis data indicated that the H3K18la and H4K12la levels in the *S6K2* promoter were significantly elevated in the WSSV‐infected shrimp hemocytes compared to the control (Figure 3H). These results demonstrated that the virus infection promoted the enrichment of H3K18la and H4K12la in the *S6K2* promoter region.

Collectively, these data indicated that 75 target genes regulated by H3K18la and H4K12la were identified, of which H3K18la and H4K12la modification activated the transcription of *S6K2* in the WSSV‐infected shrimp.

### 2.4 Role of S6K2 in Virus Infection

**Figure 4:**
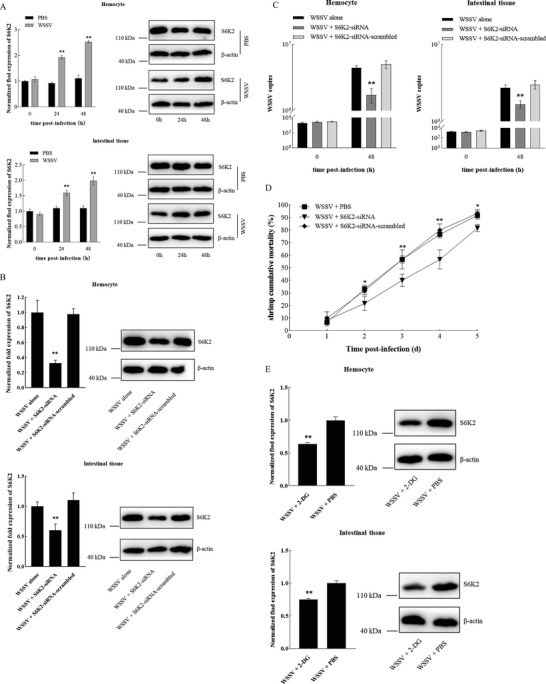
Role of S6K2 in virus infection. A) Differential expression of S6K2 in the WSSV‐challenged and healthy shrimp. Shrimp were injected with WSSV or PBS. At different time after injection, the expression of S6K2 in the hemocytes and intestinal tissues of shrimp was examined by quantitative real‐time PCR (**p < 0.01) (left) and Western blot (right). β‐actin was used as a control. B) Knockdown of S6K2 in shrimp. The WSSV‐infected shrimp was injected with S6K2‐specific siRNA (S6K2‐siRNA) or S6K2‐siRNA‐scrambled as a control. At 48 h after injection, the expression level of S6K2 in the hemocytes and intestinal tissues of shrimp was examined using quantitative real‐time PCR (**p < 0.01) and Western blot. C) Impact of S6K2 silencing on virus infection in shrimp. The WSSV‐infected shrimp were injected with S6K2‐siRNA and S6K2‐siRNA‐scrambled. At different time after injection, the virus copy in the hemocytes and intestinal tissues of shrimp was quantified by quantitative real‐time PCR (**p < 0.01). D) Effects of S6K2 silencing on the mortality of WSSV‐infected shrimp. Shrimp were co‐injected with WSSV and S6K2‐siRNA or S6K2‐siRNA‐scrambled. The numbers on the horizontal axis indicated the days post‐infection (*p < 0.05; **p < 0.01). E) Influence of 2‐DG on the expression of S6K2 in shrimp. WSSV‐infected shrimp was injected with 2‐DG. PBS was included in the injection as a control. At 48 h after injection, the expression level of S6K2 in the hemocytes and intestinal tissues of shrimp was examined using quantitative real‐time PCR (**p < 0.01) and Western blot. F) Impact of 2‐DG on virus infection in shrimp. The WSSV‐infected shrimp were injected with 2‐DG. At different time after injection, the virus copies in the hemocytes and intestinal tissues of shrimp were examined by quantitative real‐time PCR (**p < 0.01). G) Effects of 2‐DG on the mortality of WSSV‐infected shrimp. The number on the horizontal axis indicated the days post‐infection (*p < 0.05; **p < 0.01). H) Impact of LDH silencing on the S6K2 expression in shrimp. The WSSV‐infected shrimp was injected with LDH‐siRNA or LDH‐siRNA‐scramble. At 48 h after injection, the expression level of S6K2 in the hemocytes and intestinal tissue of shrimp was examined (**p < 0.01). I) Examination of virus copy in the LDH‐silenced shrimp. The virus copy was quantified using quantitative real‐time PCR (**p < 0.01). (J) Mortality of the virus‐infected shrimp. The WSSV‐infected shrimp were injected with LDH‐siRNA or siRNA‐scrambled. At different time post‐infection, the shrimp mortality was determined (*p < 0.05; **p < 0.01).

To evaluate the involvement of S6K2 in the virus infection, the expression level of S6K2 in the WSSV‐infected and virus‐free shrimp was determined. The results indicated that S6K2 was significantly upregulated in the hemocytes and intestinal tissues of shrimp (**Figure** **4**A), suggesting the important role of S6K2 in virus infection.

To explore the role of S6K2 in the WSSV infection, S6K2 was silenced in the WSSV‐infected shrimp, followed by the examination of virus copy. The results showed that S6K2 was knocked down in shrimp (Figure 4B). The S6K2 silencing led to significant decreases of virus copy in the hemocytes, and intestinal tissue of shrimp and the mortality of WSSV‐infected shrimp compared to the controls (Figure 4C,D). These results demonstrated that S6K2 could promote virus infection in shrimp.

To assess the influence of glycolysis‐mediated H3K18la and H4K12la of *S6K2* promoter on the expression of *S6K2* in shrimp, shrimp were treated with 2‐DG, followed by the examination of *S6K2* expression. The results showed that the treatment of 2‐DG inhibited the *S6K2* expression in the hemocytes and intestinal tissues of shrimp (Figure 4E). To evaluate the effects of 2‐DG on virus infection in shrimp, the WSSV‐challenged shrimp were treated with 2‐DG, followed by the examination of virus copies and shrimp mortality. The results demonstrated that the 2‐DG treatment significantly reduced the virus copies in shrimp and the shrimp cumulative mortality compared with the control (Figure 4F,G), indicating that the suppression of *S6K2* expression mediated by H3K18la and H4K12la inhibited the virus infection in shrimp. These results revealed that glycolysis‐mediated H3K18la and H4K12la promoted the *S6K2* expression, leading to the promotion of the virus infection in shrimp.

To further explore the effects of the glycolysis‐mediated H3K18la and H4K12la on the expression of S6K2, LDH was knocked down in shrimp and then the expression of S6K2 was detected. The results revealed that the LDH silencing inhibited the S6K2 expression in shrimp (Figure 4H). To evaluate the impact of LDH silencing on the virus infection, the WSSV infection was determined in the LDH‐silenced shrimp. The results showed that the LDH silencing significantly reduced the virus copy in shrimp and virus‐infected shrimp mortality (Figure 4I,J). These results demonstrated that the glycolysis‐mediated H3K18la and H4K12la might promote the virus infection in shrimp via upregulating S6K2.

Taken together, these findings presented that the glycolysis‐mediated H3K18la and H4K12la promoted the expression of *S6K2*, the target gene of H3K18la and H4K12la, thus resulting in the promotion of the virus infection in shrimp.

### 2.5 Regulation of H3K18la and H4K12la Mediated by Viral miRNAs

**Figure 5:**
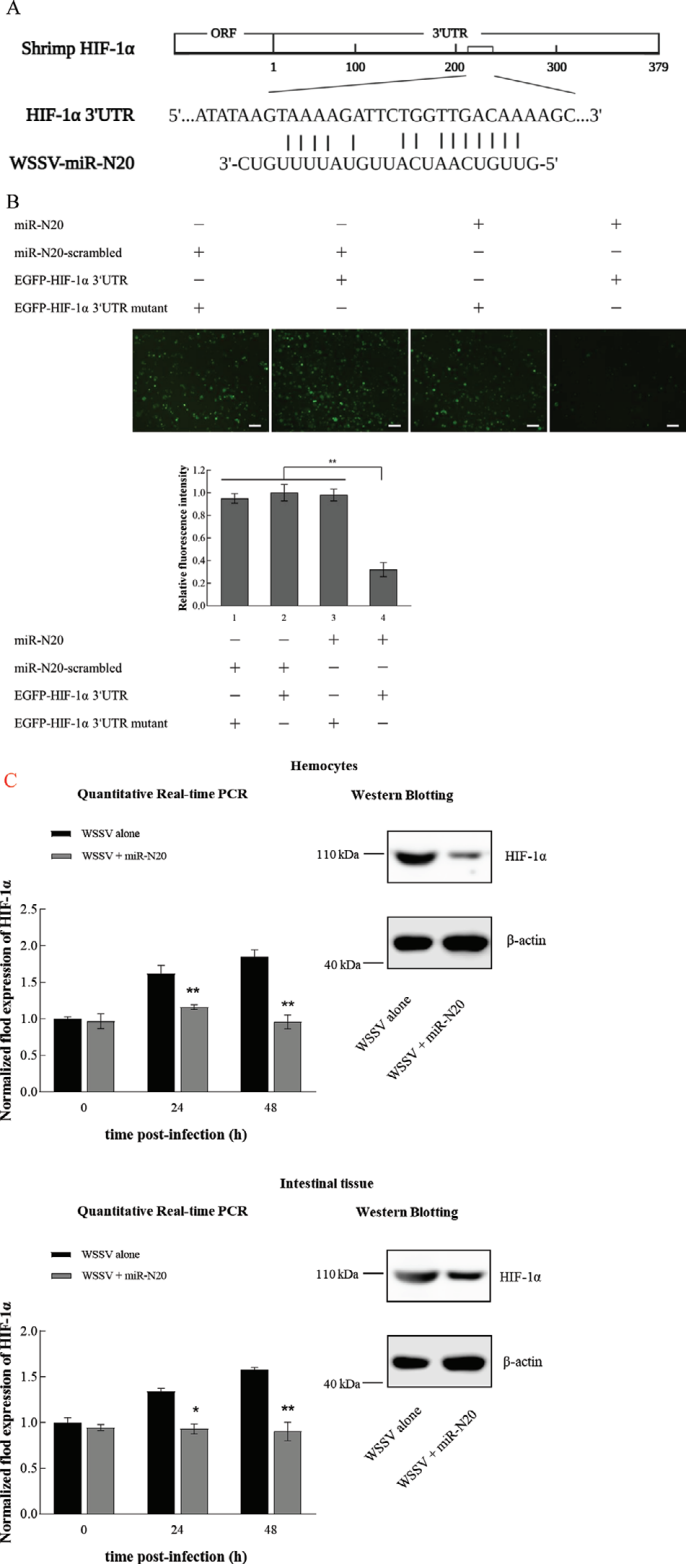
Regulation of H3K18la and H4K12la mediated by viral miRNAs. A) Prediction of WSSV miRNAs targeting hypoxia inducible factor‐1α (HIF‐1α). Based on the prediction, WSSV‐miR‐N20 could target shrimp *HIF‐1α*. The underline showed the seed sequence of miR‐N20. B) Direct interaction between miR‐N20 and *HIF‐1α*. The insect High Five cells were co‐transfected with miR‐N20 or miR‐N20‐scrambled and plasmid EGFP‐HIF‐1α 3′UTR or EGFP‐HIF‐1α 3′UTR mutant. At 36 h after tansfection, the fluorescence of insect cells was observed under a fluorescence microscope. The relative fluorescence intensity was calculated with Image J (**p < 0.01). Scale bar, 100 µm. C) Effects of miR‐N20 overexpression on the expression of HIF‐1α in the hemocytes and intestine of shrimp. WSSV‐infected shrimp were injected with miR‐N20. Forty‐eight hours later, the mRNA or protein level of HIF‐1 α was detected using quantitative real‐time quantitative PCR (*p < 0.05; **p < 0.01) (left) or Western blot. D) Impact of miR‐N20 overexpression on the LDH expression in shrimp. The WSSV‐infected shrimp were injected with miR‐N20. Forty‐eight hours later, the mRNA or protein level of LDH was detected using quantitative real‐time quantitative PCR (*p < 0.05; **p < 0.01) (left) or Western blot (right). E) Western blot analysis of H3K18la and H4K12la in shrimp overexpressing miR‐N20. The WSSV‐infected shrimp were injected with miR‐N20. At 48 h after injection, the levels of H3K18la and H4K12la in the hemocytes and intestines of shrimp were detected using Western blot. H3 was used as a control.

To explore the regulation of histone lactylation mediated by virus‐encoded miRNAs, the WSSV miRNAs targeting hypoxia inducible factor‐1α (HIF‐1α), the key regulator of glycolysis, were predicted. The results indicated that the virus‐encoded WSSV‐miR‐N20 might target shrimp *HIF‐1α* (**Figure** **5**A).

To investigate the interaction between miR‐N20 and shrimp *HIF‐1α*, pIZ/V5‐His expression plasmid containing *EGFP* and *HIF‐1α* 3′UTR was co‐transfected with miR‐N20 into insect H5 cells. The results demonstrated that the fluorescence intensity of insect cells co‐transfected with *EGFP*‐*HIF‐1α* 3′UTR and miR‐N20 was significantly reduced compared with the controls (Figure 5B), showing that miR‐N20 could target the 3′UTR of *HIF‐1α*. To explore the interaction between miR‐N20 and *HIF‐1α* in vivo, miR‐N20 was injected into WSSV‐infected shrimp, followed by the examination of *HIF‐1α* expression. The results revealed that the miR‐N20 overexpression significantly decreased the *HIF‐1α* expression in shrimp compared to the control (Figure 5C). These data indicated that *HIF‐1α* was the target gene of miR‐N20.

To further evaluate the impact of the miR‐N20‐HIF‐1α interaction on H3K18la and H4K12la, miR‐N20 was injected into the WSSV‐infected shrimp and then the expression of LDH and the H3K18la and H4K12la levels were examined. The results revealed that the miR‐N20 overexpression significantly inhibited the LDH expression level in shrimp (Figure 5D), showing that the miR‐N20‐mediated silencing of HIF‐1α suppressed the LDH expression in shrimp. At the same time, the miR‐N20 overexpression remarkably decreased the H3K18la and H4K12la levels in shrimp (Figure 5E). These results demonstrated that the miR‐N20‐HIF‐1α interaction suppressed H3K18la and H4K12la in shrimp.

Taken together, the findings revealed that the WSSV‐encoded miR‐N20 could downregulate the expression of LDH in glycolysis by targeting *HIF‐1α*, leading to the suppression of H3K18la and H4K12la.

### 2.6 Influence of miR‐N20‐MEDIATED H3K18la and H4K12la on S6K2 and Virus Infection

**Figure 6:**
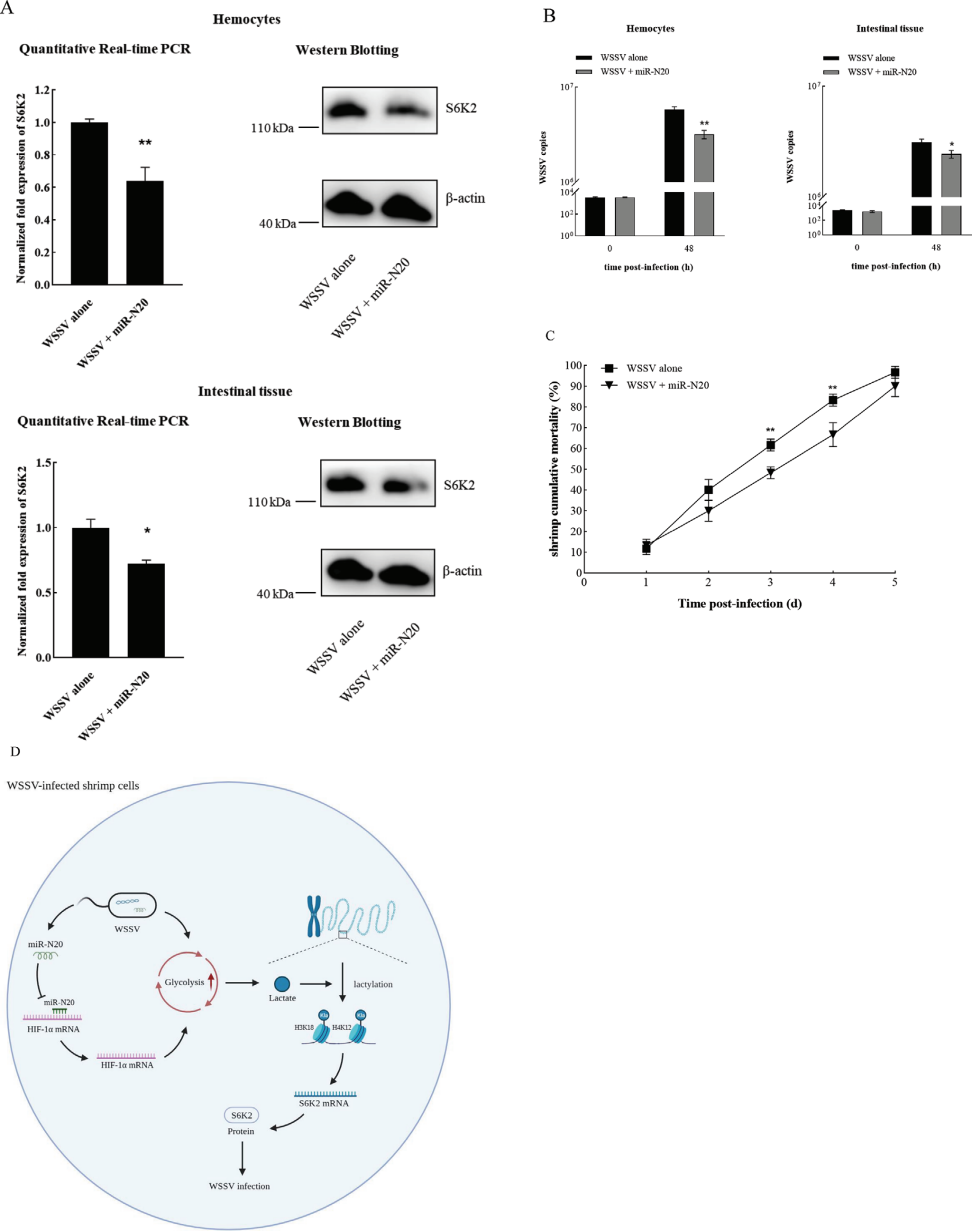
Influence of miR‐N20‐mediated H3K18la and H4K12la on S6K2 and virus infection. A) Effects of miR‐N20 overexpression on the expression of S6K2 in the hemocytes and intestine of shrimp. WSSV‐infected shrimp were injected with miR‐N20. Forty‐eight hours later, the mRNA or protein level of S6K2 was detected using quantitative real‐time PCR (*p < 0.05; **p < 0.01) (left) or Western blot (right). B) Effects of miR‐N20 overexpression on virus infection in shrimp. The WSSV‐infected shrimp were injected with miR‐N20. At different time after injection, the virus copy in the hemocytes and intestinal tissue of shrimp was quantified by real‐time PCR (*p < 0.05; **p < 0.01). C) Influence of miR‐N20 overexpression on the mortality of WSSV‐infected shrimp. The numbers on the horizontal axis indicated the post‐infection days (**p < 0.01). D) Model for the role of glycolysis‐mediated histone lactylation in virus infection.

To explore the impact of miR‐N20‐mediated H3K18la and H4K12la on S6K2, miR‐N20 was expressed in the virus‐infected shrimp and then the expression of *S6K2* was examined. The results revealed that the miR‐N20 overexpression significantly inhibited the expression of *S6K2* in compared with the control (**Figure** **6**A). These results indicated that miR‐N20 could suppress the expression of *S6K2*, the target gene of H3K18la and H4K12la.

To evaluate the influence of miR‐N20‐mediated H3K18la and H4K12la on the virus infection, miR‐N20 was overexpressed in the WSSV‐infected shrimp, followed by the examination of virus copies. The result demonstrated that the miR‐N20 overexpression significantly reduced the virus copies compared with the control (Figure 6B). At the same time, the miR‐N20 overexpression led to a significant decrease of the WSSV‐infected shrimp mortality (Figure 6C). These results revealed that miR‐N20 played a negative role in the virus infection.

Taken together, the findings indicate that the virus infection induced the enhancement of glycolysis and the accumulation of lactate in shrimp to promote the site‐specific histone lactylation (H3K18la and H4K12la) (Figure 6D). The upregulation of H3K18la and H4K12la increased the expression of S6K2 to promote the virus infection (Figure 6D). At the same time, viral miR‐N20 inhibited the expression of the HIF‐1α and the downregulation of HIF‐1α, in turn, suppressed shrimp glycolysis (Figure 6D).

## 3 Discussion

As “non‐cellular organisms” consisting of only a few components, such as nucleic acids and proteins, viruses need to infect living cells and utilize the host metabolic systems to replicate and proliferate themselves.^[^ ^20^ ^]^ Thus the virus infection must alter host metabolic pathways, one of which is the enhancement of aerobic glycolysis.^[^ ^5^ ^]^ The Zika virus (ZIKV) infection enhances the aerobic glycolysis of cells and increases the expression of glycolysis‐related genes, such as monocarboxylate transporter 4 (*MCT4*), glucose transporter 1 (*GLUT1*) and glucose transporter 2 (*GLUT2*).^[^ ^21^ ^]^ Human cytomegalovirus (HCMV) infection to human fibroblasts exhibits a notable increase of glucose consumption and induces the expression of glucose transporter 4 (*GLUT4*) in host cells, thereby enhancing aerobic glycolysis.^[^ ^22^ ^]^ In shrimp, the compound palmitamide, encapsulated in the viral particles of WSSV, binds to the rate‐limiting enzyme of glycolysis, triosephosphate isomerase (TPI), to enhance its enzymatic activity, thus enhancing the process of glycolysis to facilitate the virus infection.^[^ ^5^ ^]^ During the virus infection, aerobic glycolysis not only provides rapid energy for viral replication and assembly, but also induces the accumulation of large amounts of lactate in host cells.^[^ ^23^ ^]^ The increased lactate content ensures that the host cells are able to reduce sufficient NAD+ to NADH through an enhanced glycolysis.^[^ ^24^ ^]^ In this study, our findings demonstrated that the virus infection increased the expression levels of hexokinase (HK) and lactate dehydrogenase (LDH), thus enhancing glycolysis and the accumulation of lactate in host cells. These findings suggest that lactate, a metabolite of virus‐induced aerobic glycolysis, may play a crucial role in virus‐host interactions.

It has been discovered that lactate, previously being considered a metabolic waste of cells resulting from glycolysis, actually serves in various important physiological functions.^[^ ^25^ ^]^ Lactate can act as a major precursor in the gluconeogenic cycle, contributing to other sugar metabolic processes. It also functions as a signaling molecule to regulate cellular signaling pathways and can regulate cellular activation and antigen presentation, enabling tumor cells to evade the body's immune system.^[^ ^25^ ^]^ As reported, lactate plays a crucial role in mediating immune cell reprogramming and enhancing cellular plasticity through metabolic remodeling and epigenetic modifications.^[^ ^26^ ^]^ Lactate can exert cellular regulation with non‐metabolic functions by driving histone lactylation and directly regulating gene transcription.^[^ ^13d^ ^]^ The further studies have established that the protein lactylation plays a key role in determining cell fate, embryonic development, inflammation, cancer and neuropsychiatric disorders.^[^ ^27^ ^]^ The upregulation of histone lactylation (H3K18la), caused by the accumulation of lactate, directly regulates the transcription of genes responsible for determining cell fate.^[^ ^6a^ ^]^ The in vitro hypoxic culture conditions can reduce lactate and histone lactylation levels in mouse blastocysts, impacting the development of embryonic cells.^[^ ^28^ ^]^ The lactate treatment of non‐small cell lung (nSCLC) cancer cells enhances the proliferation and migration of nSCLC by upregulating histone lactylation in the promoters of various genes encoding metabolic enzymes.^[^ ^29^ ^]^ Histone lactylation emerges as a significant post‐translational modification with a potential role in virus infection.^[^ ^30^ ^]^ These findings suggest that the lactate accumulation by aerobic glycolysis during virus infection may play important roles in the epigenetic regulation of hosts. However, the roles and mechanisms of aerobic glycolysis and lactate in virus infection remains unclear. In this study, the results showed that the virus infection enhanced host glycolysis, leading to the lactate accumulation. The accumulated lactate in turn promoted the intracellular histone lactylation (H3K18la and H4K12la) level to upregulate the expression of target gene shrimp *S6K2*. As reported, the phosphorylation of S6K2 can enhance the WSSV infection in shrimp.^[^ ^31^ ^]^ Thereby, the upregulation of S6K2 mediated by H3K18la and H4K12la could promote the virus infection. Based on the important roles of miRNAs in the virus‐host interactions,^[^ ^32^ ^]^ the results of this study revealed that WSSV‐miR‐N20 could suppress the virus infection by targeting *HIF1a* to inhibit histone lactylation process. These data implied that the viral miRNAs played essential roles in the histone lactylation‐mediated virus infection. During the WSSV infection, the viral miRNA WSSV‐miR‐N32 targets viral mRNAs of wsv459 and wsv322, leading to the suppression of WSSV infection.^[^ ^33^ ^]^ The targeting of viral genes by viral miRNAs may result in virus latency, which is an efficient strategy for virus to resist the innate immunity of hosts.^[^ ^33^ ^]^ In this context, our findings provided novel insights into how virus manipulated cellular metabolism to facilitate the virus infection.

## 4 Experimental Section

### Crustacean Culture and Virus Infection

A crustacean shrimp (*Marsupenaeus japonicus*) ≈10 g were raised in groups of 20 individuals in 80L aquariums at 20–25 °C. In prior to experiments, three shrimp, randomly selected, were subjected to PCR using WSSV‐specific primers to exclude WSSV infection. The primers for PCR were listed in Table S2 (Supporting Information). Subsequently 100 µL of WSSV (10^5^ copies ml^−1^) were intramuscularly injected into the lateral area of the fourth abdominal segment of virus‐free shrimp with a syringe with a 29‐gauge needle. At various times post‐infection, the WSSV‐infected shrimp were collected for later used.

### Determination of Lactate Content In Vivo

The LA Assay Kit (Solarbio, Beijing, China) was used to determine the lactate content in shrimp according to the manufacturer's protocol. Shrimp hemolymph was centrifuged at 300×g for 5 min to collect hemocytes. The hemocytes or intestinal tissues were added with 1 ml of cell extract I, follows by ultrasonication on ice for 3 min. After centrifugation at 12 000×g for 10 min at 4 °C, the supernatant was added with cell extract II and then centrifuged at 4 °C for 10 min. The absorbance of the supernatant was determined at 570 nm. At the same time, the standard curve was plotted. The lactate content was calculated.

To evaluate the role of glycolysis in shrimp, shrimp were intramuscularly injected with 36 mM of 2‐deoxy‐D‐glucose (Sigma–Aldrich, USA) every 24 h to inhibit glycolysis. At different time after the first injection (24, 36, and 48 h), the content of lactate was examined.

### Quantification Analysis of mRNAs by Quantitative Real‐Time PCR

The total RNAs were extracted from shrimp hemocytes or tissues with a commercial kit (Ambion, USA). After treatment with DNase I, the cDNA was reversely transcribed using PrimeScript First Strand cDNA Synthesis Kit (Takara, Japan). The quantitative real‐time PCR reaction mixture (10 µL) contained 5 µL of SYBR Green PCR Master Mix (Takara, Japan), 0.4 µL of 10 µM forward and reverse primers, and 1 µL of cDNA. The primers were gene‐specific. The shrimp β‐actin gene was used as a control. PCR was performed at 95 °C for 10 min, followed by 50 cycles at 95 °C for 15 s and 60 °C for 1 min. The gene identity (ID) and primers for quantitative real‐time PCR were listed in Tables S1 and S2 (Supporting Information).

### Detections of Hexokinase and Lactate Dehydrogenase Activities

The activity of hexokinase or lactate dehydrogenase was determined using a commercial kit according to the manufacturer's protocols (Solarbio, Beijing, China). Shrimp hemolymph was centrifuged at 300×g for 5 min to collect hemocytes. The hemocytes or intestinal tissues were added with cell extract buffer, followed by ultrasonication on ice. After centrifugation at 8000×g for 10 min at 4 °C, the supernatant was added with reaction buffer. The absorbance was measured at 340 nm to determine hexokinase activity or at 450 nm to determine lactate dehydrogenase activity. The experiments were performed in triplicate for each sample.

### Quantification of WSSV Copies

The genomic DNA was extracted from WSSV‐infected shrimp hemocytes using a DNA isolation kit according to the manufacturer's protocol (Generay, Shanghai, China). Then the extracted genomic DNA was subjected to quantitative real‐time PCR using WSSV‐specific primers and TaqMan probe (Table S2, Supporting Information). A linearized plasmid containing a 1400 bp DNA fragment of WSSV genome was quantified as an internal standard.

### Internalization Assay with Fluorescein Isothiocyanate (FITC)‐Labeled WSSV

FITC (Sigma‐Aldrich, USA) was used to label the purified WSSV virions for 1 h at room temperature. After washes with 0.1 M NaHCO_3_ (pH 9.0), the FITC‐labeled WSSV virions were mixed with the shrimp hemocytes a ratio of 50:1. Subsequently the mixture was incubated at 28 °C for 30 min. After washes three times with phosphate buffered saline (PBS), the fluorescence of un‐phagocytosed FITC‐labeled WSSV was quenched with the trypan blue solution (Amresco, USA). The hemocytes were fixed in 1% paraformaldehyde (Sigma–Aldrich, USA) at room temperature for 10 min, followed by washes with PBS and observation under a fluorescence microscope. A total of 500 hemocytes were examined for each treatment and then the phagocytic percentage was determined. The experiment was biologically repeated for three times. The data were presented as mean ± standard deviation.

### Western Blot Analysis

The concentration of the extracted proteins was determined using Bradford assay (Beyotime Biotechnology, Shanghai, China). The proteins were separated with SDS‐PAGE and then eletrotransferred to a polyvinyl difluoride membrane (Millipore, USA). After incubation with blocking solution [5% skim milk in TBST (Tris buffered saline with Tween 20)] for 2 h at room temperature, the membrane was incubated with a primary antibody at 4 °C overnight. Subsequently the membrane was incubated with horseradish peroxidase (HRP)‐conjugated secondary antibody (Bio‐Rad, USA) for 2 h at room temperature. The proteins were examined using Western Lightning Plus‐ECL Oxidizing Reagent Plus (PerkinElmer, USA). All the primary monoclonal antibodies against histones H3 and H4 were purchased from PTM BIOLABS (Hangzhou, China). The antibodies against shrimp HIF‐1α, LDH and S6K2 were prepared in this study. The primers for vector construction were listed in Table S3 (Supporting Information).

### Silencing of Target Gene In Vivo

To knock down the expression of a target gene in shrimp in vivo, the target gene sequence‐specific siRNA (30 µg shrimp^−1^) was intramuscularly injected into the lateral area of the fourth abdominal segment of shrimp using a syringe with a 29‐gauge needle. As a control, siRNA‐scrambled (30 µg shrimp^−1^) was included in the injection. Twelve hours later, the shrimp were re‐injected with the same siRNA (30 µg shrimp^−1^) or siRNA‐scrambled (30 µg shrimp^−1^). At different time points after the last siRNA injection, three shrimp were randomly collected for later use. The siRNAs (Table S4, Supporting Information) were synthesized using the T7 High Yield RNA Transcription Kit (Vazyme, Nanjing, China) according to the manufacturer's protocol.

### Treatment of Shrimp with Compounds

To suppress or enhance glycolysis or histone lactylation of shrimp, shrimp were intramuscularly injected with 100 µl of 2‐deoxy‐D‐glucose (2‐DG, 36 mM), sodium lactate (Nala, 5 M), Vorinostat (SAHA, 2 mM) or C646 (30 mM) (sigma–Aldrich, USA) in the lateral area of the fourth abdominal segment using a syringe with a 29‐gauge needle once every 24 h. As a control, PBS or DMSO was included in the injections. The concentrations of the compounds used were determined based on the previous study.^[^ ^5^ ^]^ 2‐DG, a glucose analog, is an inhibitor of glycolysis,^[^ ^14^ ^]^ while Nala, the product of glycolysis, can promote histone lactylation.^[^ ^15^ ^]^ SAHA is a potent pan‐inhibitor of histone deacetylase^[^ ^16^ ^]^ and C646 is a competitive inhibitor of histone acetyltransferase p300.^[^ ^17^ ^]^ At different time after injection, the hemocytes of intestinal tissues of shrimp were collected for later.

### CUT&Tag Analysis

CUT&Tag analysis was performed using the hyperactive universal CUT&Tag assay kit for Illumina (Vazyme Biotech, Nanjing, China) according to the manufacturer's protocol. The shrimp hemocytes were bound to ConA beads (Vazyme Biotech) and then resuspended in the antibody buffer (Vazyme Biotech) to increase the hemocyte permeability. The hemocytes were incubated with the antibody against H3K18la or H4K12la (PTM Bio, Hangzhou, China) at 4 °C overnight, followed by incubation with the secondary antibody against rabbit IgG (Beyotime, Shanghai, China) for 1 h at room temperature. After incubation with pA/G‐Tnp transposase (Vazyme Biotech) for 1 h at room temperature, DNA was extracted from the sample to construct the library. The library was quantified with dsDNA 915 Reagent Kit (Agilent, USA) and sequenced by Illumina novaseq (Illumina, USA). The deepTools was used to analyze and visualize the CUT&Tag results.

### Chromatin Immunoprecipitation Assay (ChIP)‐Quantitative Real‐Time PCR (qPCR)

ChIP was performed using the ChIP assay kit (Beyotime Biotechnology, Shanghai, China) with the anti‐H3K18la or anti‐H4K12la antibody (PTM Bio, Hangzhou, China). Briefly, the isolated hemocytes were fixed with 1% formaldehyde for 10 min at room temperature and then resuspended in the SDS lysis buffer containing protease inhibitors, followed by sonication for 8 min on ice. The sonicated chromatin fragments were diluted by ChIP dilution buffer containing protease inhibitors. For immunoprecipitation, the mixture was briefly incubated with mouse IgG (control), anti‐H4K12la or anti‐H3K18la antibody overnight at 4 °C. Protein A+G agarose was added into the supernatant for an additional 1 h at 4 °C. The pulled‐down chromatin complex was released after dilution with 100 µl of 0.2 M glycine. The DNA was purified with the DNA purification kit (Beyotime Biotechnology). Fold enrichment was quantified using qRT‐PCR.

### Kyoto Encyclopedia of Genes and Genomes (KEGG) Analysis

The genes were subjected to pathway enrichment analysis to identify the significantly enriched biological pathways. This analysis was performed based on KEGG pathway database (https://www.genome.jp/entry/gn:T07703). The pathways were identified to be significantly enriched when false discovery rate (FDR) was less than 0.05.

### Transcriptome Analysis

Shrimp were injected with WSSV or PBS. At 48 h after the injection, shrimp hemocytes were collected to extract the total RNAs using the Trizol reagent (Thermo Fisher Scientific, USA). Then the libraries were constructed from the total RNAs using NEBNext UltraTM RNA Library Prep Kit (New England Biolabs, USA). Sequenced was carried out using Illumina Novaseq 6000 platform (Illumina, USA). The differentially expressed genes were identified using the criteria of the false discovery rate (FDR) <0.01 and fold changes <0.5 or >2.0 (<− 1 or > 1 log2 ratio value, *p* value <0.05).

### Prediction of miRNAs Targeting a Gene

To predict the miRNAs that could target a gene, the algorithm RNAhybrid was used. The online prediction (http://bibiserv.techfak.uni‐bielefeld.de/rnahybrid) was performed based on the binding of a miRNA to the 3′ untranslated region (3′UTR) of a gene.

### Interaction between WSSV miR‐N20 with HIF‐1α

Insect high five cells were cultured in Express FiveSFM medium (Gibco, USA) containing 10% L‐glutamine (Invitrogen, USA). When the cell density was greater than 70%, the pIZ/V5‐His recombinant plasmid with wild‐type 3′UTR or mutant 3′UTR of the target gene HIF‐1α (Supporting information of sequences) was co‐transfected into the cells with 100 pM miR‐N20 mimic or miR‐N20‐scrambled using lipofectamine 2000 (Invitrogen). The cells were cultured at 27 °C for 12 h and then the medium was changed. At 36 h after transfection, the fluorescence value of cells was measured with a microplate chemiluminescence detector GloMax96 (Promega, USA) at 490 nm/510 nm.

To examine the interaction between miR‐N20 and HIF‐1α in vivo, miR‐N20 (5′‐GUUGUCAAUCAUUGUAUUUUGUC‐3′) was synthesized using the in vitro transcription T7 kit (Vazyme Biotech, Nanjing, China) and injected into shrimp at 30 µg shrimp^−1^. At different time after injection, the shrimp hemocytes and intestinal tissues were collected to detect the expression level of HIF‐1α.

### Shrimp Mortality

Shrimp were subjected to different treatments (20 shrimp per treatment). At various time points (1, 2, 3, 4 and 5 days) after treatment, the cumulative shrimp mortality was evaluated.

### Statistical Analysis

One‐way analysis of variation was used to calculate the means and standard deviations (SD) in all data gathered from at least 3 independent experiments. Significant differences between treatments were determined using Student's t‐test. GraphPad Prism 8 software (GraphPad Software Inc., USA) was applied to perform statistical analysis.

## Conflict of Interest

The authors declare no conflict of interest.

## Author Contributions

Y.Z. and X.B.Z. designed the study. Y.Z. performed the experiments. X.Z. wrote and edited the manuscript. All authors discussed the results and commented on the manuscript.

## Supporting information

Supporting Information

## Data Availability

The data that support the findings of this study are available from the corresponding author upon reasonable request.

## References

[1] a) M. Zhao , H. Zhang , K. Liu , G. F. Gao , W. J. Liu , Sci. China Life Sci. 2017, 60, 1307; 29294219 10.1007/s11427-017-9241-3 PMC7089170

[2] a) Y. Wang , P. Wang , Y. Zhang , J. Xu , Z. Li , Z. Li , Z. Zhou , L. Liu , X. Cao , Immunity 2020, 53, 1168; 33326766 10.1016/j.immuni.2020.11.010

[3] N. S. Heaton , G. Randall , Cell Host Microbe 2010, 8, 422. 21075353 10.1016/j.chom.2010.10.006 PMC3026642

[4] a) N. S. Heaton , R. Perera , K. L. Berger , S. Khadka , D. J. Lacount , R. J. Kuhn , G. Randall , Proc. Natl. Acad. Sci. U S A 2010, 107, 17345; 20855599 10.1073/pnas.1010811107 PMC2951450

[5] S. Zhang , F. Xin , X. Zhang , iScience 2021, 24, 101915. 33385116 10.1016/j.isci.2020.101915 PMC7770649

[6] a) S. Atlante , A. Mongelli , V. Barbi , F. Martelli , A. Farsetti , C. Gaetano , Clin. Epigenetics 2020, 12, 156; 33087172 10.1186/s13148-020-00946-x PMC7576975

[7] G. Yang , J. Feng , Y. Liu , M. Zhao , Y. Yuan , H. Yuan , H. Yun , M. Sun , Y. Bu , L. Liu , Z. Liu , J. Q. Niu , M. Yin , X. Song , Z. Miao , Z. Lin , X. Zhang , Theranostics 2019, 9, 7345. 31695772 10.7150/thno.37173 PMC6831306

[8] G. Z. Liu , X. W. Xu , S. H. Tao , M. J. Gao , Z. H. Hou , J. Biomed. Sci. 2021, 28, 67. 34615538 10.1186/s12929-021-00762-2 PMC8495979

[9] Y. L. Wu , D. Wang , X. E. Peng , Y. L. Chen , D. L. Zheng , W. N. Chen , X. Lin , Free Radic. Biol. Med. 2013, 65, 632. 23920313 10.1016/j.freeradbiomed.2013.07.037

[10] S. Liu , L. Liu , G. Xu , Z. Cao , Q. Wang , S. Li , N. Peng , J. Yin , H. Yu , M. Li , Z. Xia , L. Zhou , Y. Lin , X. Wang , Q. Li , C. Zhu , X. Yang , J. Wang , Y. She , M. Lu , Y. Zhu , J. Virol. 2019, 93, 01587. 10.1128/JVI.01587-18 PMC6430541 30651365

[11] P. C. Wu , Y. H. Lin , T. C. Wu , S. T. Lee , C. P. Wu , Y. Chang , Y. L. Wu , Sci. Rep. 2018, 8, 17817. 30546025 10.1038/s41598-018-35782-w PMC6292938

[12] S. Li , F. Ma , T. Yokota , G. Garcia, Jr. , A. Palermo , Y. Wang , C. Farrell , Y. C. Wang , R. Wu , Z. Zhou , C. Pan , M. Morselli , M. A. Teitell , S. Ryazantsev , G. A. Fishbein , J. T. Hoeve , V. A. Arboleda , J. Bloom , B. Dillon , M. Pellegrini , A. J. Lusis , T. G. Graeber , V. Arumugaswami , A. Deb , JCI Insight 2021, 6, 145027. 33284134 10.1172/jci.insight.145027 PMC7934846

[13] a) X. Li , Y. Yang , B. Zhang , X. Lin , X. Fu , Y. An , Y. Zou , J. X. Wang , Z. Wang , T. Yu , Signal Transduct. Target Ther. 2022, 7, 305; 36050306 10.1038/s41392-022-01151-3 PMC9434547

[14] Z. Zhu , W. Jiang , J. N. McGinley , H. J. Thompson , Cancer Res. 2005, 65, 7023. 16061689 10.1158/0008-5472.CAN-05-0453

[15] Y. Xie , H. Hu , M. Liu , T. Zhou , X. Cheng , W. Huang , L. Cao , Front. Genet. 2022, 13, 949252. 36081996 10.3389/fgene.2022.949252 PMC9445422

[16] V. M. Richon , S. Emiliani , E. Verdin , Y. Webb , R. Breslow , R. A. Rifkind , P. A. Marks , Proc. Natl. Acad. Sci. U S A 1998, 95, 3003. 9501205 10.1073/pnas.95.6.3003 PMC19684

[17] E. M. Bowers , G. Yan , C. Mukherjee , A. Orry , L. Wang , M. A. Holbert , N. T. Crump , C. A. Hazzalin , G. Liszczak , H. Yuan , C. Larocca , S. A. Saldanha , R. Abagyan , Y. Sun , D. J. Meyers , R. Marmorstein , L. C. Mahadevan , R. M. Alani , P. A. Cole , Chem. Biol. 2010, 17, 471. 20534345 10.1016/j.chembiol.2010.03.006 PMC2884008

[18] F. Zhu , X. Zhang , Sci. Rep. 2013, 3, 2069. 23797713 10.1038/srep02069 PMC3691566

[19] M. J. Richer , L. L. Pewe , L. S. Hancox , S. M. Hartwig , S. M. Varga , J. T. Harty , J. Clin. Invest. 2015, 125, 3477. 26241055 10.1172/JCI81261 PMC4588296

[20] K. Girdhar , A. Powis , A. Raisingani , M. Chrudinova , R. Huang , T. Tran , K. Sevgi , Y. Dogus Dogru , E. Altindis , Annu. Rev. Virol. 2021, 8, 373. 34586876 10.1146/annurev-virology-091919-102416 PMC9175272

[21] S. Singh , P. K. Singh , H. Suhail , V. Arumugaswami , P. E. Pellett , S. Giri , A. Kumar , J. Immunol. 2020, 204, 1810. 32086387 10.4049/jimmunol.1901310 PMC7310572

[22] Y. Yu , T. G. Maguire , J. C. Alwine , J. Virol. 2011, 85, 1573. 21147915 10.1128/JVI.01967-10 PMC3028904

[23] L. Zhou , R. He , P. Fang , M. Li , H. Yu , Q. Wang , Y. Yu , F. Wang , Y. Zhang , A. Chen , N. Peng , Y. Lin , R. Zhang , M. Trilling , R. Broering , M. Lu , Y. Zhu , S. Liu , Nat. Commun. 2021, 12, 98. 33397935 10.1038/s41467-020-20316-8 PMC7782485

[24] I. T. Chen , D. Y. Lee , Y. T. Huang , G. H. Kou , H. C. Wang , G. D. Chang , C. F. Lo , Sci. Rep. 2016, 6, 27732. 27279169 10.1038/srep27732 PMC4899751

[25] N. Li , Y. Kang , L. Wang , S. Huff , R. Tang , H. Hui , K. Agrawal , G. M. Gonzalez , Y. Wang , S. P. Patel , T. M. Rana , Proc. Natl. Acad. Sci. U S A 2020, 117, 20159. 32747553 10.1073/pnas.1918986117 PMC7443867

[26] B. S. Ferguson , M. J. Rogatzki , M. L. Goodwin , D. A. Kane , Z. Rightmire , L. B. Gladden , Eur. J. Appl. Physiol. 2018, 118, 691. 29322250 10.1007/s00421-017-3795-6

[27] X. Liu , Y. Zhang , W. Li , X. Zhou , Front Cell Dev. Biol. 2022, 10, 972020. 36092712 10.3389/fcell.2022.972020 PMC9462419

[28] W. Yang , P. Wang , P. Cao , S. Wang , Y. Yang , H. Su , B. Nashun , Epigenetics Chromatin 2021, 14, 57. 34930415 10.1186/s13072-021-00431-6 PMC8691063

[29] J. Jiang , D. Huang , Y. Jiang , J. Hou , M. Tian , J. Li , L. Sun , Y. Zhang , T. Zhang , Z. Li , Z. Li , S. Tong , Y. Ma , Front. Oncol. 2021, 11, 647559. 34150616 10.3389/fonc.2021.647559 PMC8208031

[30] Y. Pang , Y. Zhou , Y. Wang , L. Fang , S. Xiao , J. Virol. 2024, 98, e0167023. 38088561 10.1128/jvi.01670-23 PMC10804950

[31] P. P. Hong , C. Li , G. J. Niu , X. F. Zhao , J. X. Wang , PLoS Pathog. 2022, 18, e1010808. 36067252 10.1371/journal.ppat.1010808 PMC9481175

[32] a) Y. He , X. Zhang , RNA Biol 2012, 9, 1019; 22832246 10.4161/rna.20741

[33] Y. He , T. Ma , X. Zhang , Front. Immunol. 2017, 8, 1546. 29230209 10.3389/fimmu.2017.01546 PMC5712064

