# Supplementary material for: Virus‐Induced Histone Lactylation Promotes Virus Infection in Crustacean
Source: Adv Sci (Weinh). 2024 Jun 14;11(30):2401017. doi: 10.1002/advs.202401017 (PMC11321649; doi:10.1002/advs.202401017)

**Supplementary information of sequences**

**1) The sequence of shrimp HIF-1α mRNA**

5’-GTCAGTCGAACCCGGGTTAGCAGCTTGGAGAGCCACGTCCCGTCATCGAAGGATTTTATATTAATCAAGCGAGCAATTATCGTACTTGGTAACTTTCTTTGAACTTTCACATTCTGACAACAACAGCAAGTATCGTTTGCCTGTGACAAGTGATAAAGACTTATCGGAAAAGATAAAGTGAGTGATATTTATAGATATATATTTATATAAATATAGACACATATCTGTCGCAGCGGTTGAGAGGAGACACTACATGTGACTTGCAAGAGTATGTGCTTAAGGACTAATTTTGAAGCTGTTGGCAGGGGCGCTCGACCCTTCAAACGAGCCAGCGCAAACTCCGCAAAAGCCCAGAAGAACAGCGAGAAGCGCAAGGAGAAGTCTCGCGACGCGGCGAGATGTCGGCGAGGCAAAGAGAGCGAGATCTTCACGGAGCTGGCGAGCGCCCTGCCCTTGCCGGCGCAGACGGTGTCGCAGCTGGACAAGGCATCTGTCATGCGGCTTACCATTGCCTACCTGAAGACGCGGGCACTGTGCCAGGGCGGATTCCCGAAGATCACGGAAGGAGGCTCCTGCGCGAGTAACGGCGGCGGCGGGGGCAAGATGGACGTCGAGATGGACACCCTCTTCCTGAAGGCGCTGGACGGCTTCCTGCTGGTGCTCTCCACGGACGGCGACGTCGTCTACACGTCCGAGAACATCGTCATTTTCCTCGGCCTCTCGCAGGTGGACGTGATGGGACAGTCGCTCTACGAGTACACCCACCCGTGCGACCACGAGGAGGTGCGGGAGCTGGTGTCCGCCAAGGGCCCTCAGGAGCCCCGCCACGCCTTCCTCAGGCTCAAGTGCACGCTCACGGCGAAAGGACGCAGCGTCAACCTCAAGAGCGCTTCCTATAAGGTGGTGCAGGTGAGCGGCGAGGTGGTGCAGCACGAGGAGGACCAGACCTGGCTGGTGGCACTAGGCACCCCCGTGCCTCACCCTTCCAACATTGAATTCCCGCTGGATAAGCAGACCTTCGTCAGCAAGCACTCCCTGGACATGAAGTTCACTTACGTGGATGACAACGTGGGGGAATTCTGCGGCTACGGGCCCAAGGAGCTGGTGGGTCGCTCGCTCTACGAGATGCACCACGCGCTCGACTCCGAGCTGGTCAAGGATGCCTACAAGACACTGCGTAGCAAAGGCCAGGTGGAGACCGGGCAGTACCGGTTCCTGGCGAGAGGCGGAGGCTACGTGTGGCTGGTGACGCAGGCCACGCTCATCCACGGACCCAAGGACCACAAGCCCCAGTACGTCGTGTGCCTCAACTACGTCGTCAGTGGTGTAGAGTCTCCAGGAGAGATTTTATCAGAACTGCAATTGATGTGCAGCAAGAGCAGCAGCACCAACAGCAGCAAAGAGCCAGACAGCACTTGCAGCAAGGTGGAGAGCAGCTCCCCGCCAGTCAGCATTTCATCTCCCACTGTGGCTCCCAGTGTTCCCACACCCACGCTCCCTCTGCCCAAGTCTCCTACACAATCCAAGACTCCGCGTGTGACACCAGCGGGCCCGCCTCCAACACCTGTGGCTACCACATCCAAGATCTTCGCTCCTCGCACTGAGGAAATGAACAAAGGATTCTTGACGTTCTCTGAAGATGATCCACAGTGCACAGTGTTGAAGGAAGAACCCGAAGACTTGACCCACTTGGCTCCGTCGGGGGGTGACACGTGCGTGCCACTGCCTGCGTTCATGCCCAACCTGGATGATATGTTCTCATTTGACTACGGCCAGATACCCATTTCTACCACTGATGTCCTCTTCACCACTGCCTCATCTGTGTCCGAAGAGCAGGAGAGCAACTCGGGTTACGAGAAGAAGTTAATCAGTGACGAGAAGCGTATTAGCAGCAGGCTAAGTGGAGGGAACGTGATTATCAACAACAGCATTGGAAACAGTCCAGCGTCCAGTTGCGGCAGCCCGCACCCAGCTGGTCTCCGCACGCCTGAGCCCCCAAAACCCCTGCTGTCTCAAGCTGCTTTGCCCTCAATCCTGGAAAAGAAGATCAATTTTGGGAACTTGGACTGTAGTAGTCGCCCCAGAACAACAACAGAGAGTTTCTTCTCACAACTGGATGAGAATTCCAACCCTGGCACAGAGTTCACGAAATTGGACCTCAAGATGGAAGACCAGAATATGGACTCGGATGAATTTGACATGAGGGCTCCATACATACCCCATAGCGACGAGATGCTGGTGCTGAGCTCAGATGATCTGTTGTGGGGTGCAGAGGTAGAGCCTGCTGTATCTCCGAAGAATCAGCTTGGATACCACAAAGACACCAAGAATTTCACTCTGAACAGCAAAGAAGATTCAAGTCTTGCTCAGCTTCTGCGAGATGCTGACCCACCCATCACCAGCTGTCACACAGGGAAAGATAGTAAAGATGACAACGGAGGGGGCTCTCGGCAGAATCAGTATGAACAGAACAAGTTCTTTGACGGAGGAGAGAACTTTGTTGATCCAAACAAGGTACTTCCAGGGCACTGTATTGGAAAAGATGGTTTAGACGGATCCCCAGGGTGTAACGACCAGGGTGAAGACGAGCCACCGGCCGTAATGGTGCAGGAGACAGTTGAGCCGCCACCGCCTCTGATCACCATAGACACGAACCAGATGTCCCTGGCTGTCAAGAGGGGACTGTCTCCCAACTCGTCTCCAATCCTCACCCAGAAAAAGCTATGCTCCCTCTCGTTTCGCCAGCGACAGATGCTCTCATCTCAAGCAAGTGGGGACGGGCACTTGGCACTCCTGCAGCCTCAGCAGCAGCAGGCAGGTGGAGTGCGCCTGCTGACCACACCTTATGCCCCCACGATGCAGCAGCTGCTGATCAGCAAAGAGCCCATCACAGTGCGGGGAAGACATTCGGGAGGCCTCACAGCCCATCAGAGTTCAAACAATGACAGTAGACACTCAGTGCTTCGAAACCTGCTGGACGTGGATGAAATTGGAAGCATAGCGAGACGCGAGCCACAACCCGGAGGGAGCGGAGTGAGCTCTGCAACGTCACGTCCCTCGCAGGACAGGATGACTGACATGGCTGGCACGGGAGGCAGTGAAACCAACTCGGGCAACCATCCATCTACCCCAAAACTGAGGCTGGTCACAAGCAGCCAAGGGACGCTGATGCATGCCGGGCATCTTGCCATCAAGGTCGCTTCGAAGTCCTCGGGAGATGGCTTGTTCAAGCGAGTGCGTCGCCAGGACCCGTTGCTCCTGATGGACCCTGACCTGTCGATTCCAGACCTGCTGGATCTTACACAATTAGACTATGAGGTCAATGCTCCTGCCAATAACTGTAGTCTACTCCAGGGATCTGACCTGCTCATGGCATTAGATCAGAGTCCATAAAGTTTCTTGTTCAGTGAACATAGATTTGTGTTGAGGGAACATCCATTTTCGTAGTTTCTTAGAAAGGATGAAGATCATTGCTTTTTAGATTTTGAATGAATGATAAGTATTTTTTTAGCTCTGAAATTTTACCTTACTTCCCCTTCTTTGTGGCCAATATGTTGTTGAAGTGAAGGGATGTAAGGAATATAAGTAAAAGATTCTGGTTGACAAAAGCTCTTGGGCTTTGGAATTCCAGGAGGTCTGAAACTTGCTTAATACCATGTTCTGATTTGTCATTTACCTCTCCTTGTTATTCGGATATCCCAAGTTTGGCTGAAGTGCTGTCATGATATTGTCTTTAATGTCTTTACAGTTTAGGAGTAAAAAAAAAAAAAAAAA-3’

**2) The peak sequence of H4K12la at the S6K2 promoter site**

5’-ATCTAATACTGTATAGGGAGAGTGTCAGACTACTATGTATCTATTGTACCGCACTTTGTTTACTGCCCATAGAGTATCTATTCTCTACCAGACTGTTCGGTTCCTTCGTTTATATCCCGTAGAGGAGGTTTGAGGTCTCACTGTTGTGTTTACAGGCGGAGAGTGAGGTGTTCGGACCGGGATTTTGCCTGACACTTCTCGGAGGTATTGTGGAGTCTATACGAGGCATGCCGTTAGCCCAACAGCCGGACTTGTGGTCCAAACTGCAAGTTCAGAAGGATGATGAGGTCAGTTGGCGGAGTCTTTTTTGTGACAATAGGAGAGTGTTTCGTAGCCCAGCCCCCTCCCGGGCAGCCGCCGCCGCAACCACCTGTGTCTCTCCTAGGCTCTCTTACGCCCGTTTACGCCTGCACTGGGCTTGGGCGAGTCTCTCAGGGCTCAGGGGCTGGGGCTGGTTGGCTTGGCTCCGGTGGGGGTCCTGTCATGGCAGGGAGAGGGCAGGGGCAGGGGCAAGGGAGGGCAGCGGTGGCTGTCCTAGATGACCCAAGGCCGAGGGACGCCTGATGACCCTGATCTCGTCCCTGTCCCTTGGTGTCTGTCTGTGTGTCTGTCTGCGTGTGCTTGCGGGCGCGAGACTGCTCTTTGTTTCACAGGCGAGTGGGTGTCTGGCTGTGTTGAGACACTGTTGTGATTTGAATGCCTGCCTTCGATGCCTCCTTCGTTGCACGCATTGCAAAAGGGGAAGACGGTTGATTTTCAGATTGCGAGTTTTGCATGCTGGCAAATGATACAGACATAAATAAATAGTTTATAACCAATATAGCACATAATATATGTGTAGACTAAGGTTACATTTGATGTCATAGATTGATAAAGAAAGGATTGGTTTCCTGTCTGTTGATAAGAATGTTTCATTTTATAATGGGCCTTATGTCATTTTCTTGTTTATGACATTGCTTCTACTTGTACTTGAGGAAAAGCAGATAAATTGGTTTATGTGAATTATAAAGGATGATAACATATGAAAAAAAAGTTAACATCACATTTTCATAGACAGTATCAAGTTGAGTAGGAAAATAATGAGTATTGCAAATTGTATTTTGTTATTGTTGATATGACTTTCTGATACACAGAAGAGGATATGGAGAGAGGATGGGTACTTGATAAGGAAATTACATTGCATTTGGGTCATAGTGACGCAATCTTCGTGATAAATGACTTTCACGTCATGTACTTGATAAGCATAAGAGGATGTTTGTCTCTCTTGCAAGAAACAGATTAGAAGTCTTAGTGCTAGGAGTGTTCATGATGATTAAGTAGCAGTTGAAGCAGTGTGGTAGTCTTACTTCTTTATTGTGATAAGTTTTAACAGAAAATATTCCTTATAACACAGACACTACATTGCATTATGCACAAATAAGTAGACTTTATAACTAGGGGATTAGAATAGTTATTTTAATTGAGAGAAGTTCATTATTTAGAAGGGGGAAAATGTAAAGAGCAAATCATCTATACGCTTATTGAGGTTTTCTTCTGGAAAAGAGACATCTAGCAGCACTGGTATATAACAGAGTGCAAAGTTGTGTAATCTGCCATTTTACCTTTGTTATGGTGTTGTGCAGATACACCTATGGTAAAAGTTCTTAGATACTTTGAACTTTCTATGATAATGCTGTTAGTACAGTAGCCTTGATTTGAGAATTTTGAATATTACTTTATTTGTTTAATTTTTGAAAAGTTAGGCTGATTTCAATGACAGGTGTGGATCTCAGATGTGTACAGATAGGGAAGATTAAAGCCCTAAGTATCGCAAAGTATTTACATGGCATTGTATTTTTTTTAGTGAAGGAATATGTTATGAAATGTTTATTTTTGTTGATTGATATCATTTTGTTGATTTTTTCAAAGGTGGATTGATTGATATATATTTATATATATATATATATATATATATATATATATATATATATATATATATATATATATATATATACATATATATACATATATATAAGTATGTAATGTACATATTGATGCGCTTACTCATATATTTCTATATTGATACATATATTTGTTTATATATTTATTAATGTTATTATAGGTATTGACATTATGTAATTATTGGTATATATATATTTTTTTTTATGTAATATGCATGTATACATCTATTTGTATGTATGCATTTATTCATAGTTTAGATACAAAGTAATTAAAGATACTGAATATGTTTTTCCCTTGAATTATATAACTGACGCAGATTTGTTGTAATTACACTCCTTATCCTGCTGCAATGCATTGTCATTTAATGTTGAGTAATGGAATCAGGCATTAGTGTAGTAGACTATATAGTATTTATTTTATTATCTTTAGAAGTAATAGATTTTATCAGAGATATAACCAGGGTTTTATTTTAGTTATGATTGCTTGTTAGGGATTTGATATCTTGAGAGATTAAGATAAGAGAATCAGGATTTCCTTTTTTTGAGTACATTACACTTGGTATAAGAATGCATAAGTGAATCACATTTGTGTGTGTGTGTGTGTGTGTGTGTGTGTGTGTGTGTATCTGTGTATCTGTGTTTGTGTTTGTGTGTGTTTGTTTGGAGTGTTGAGGGGAGGGAGGGTTCTGTAGGTCTGTAATTCTGTTGTTTTCCTTTAATCCATATAGGAAATTGCAATGCAGTTTTCTGATTGTATTTCTATTCAAAGCTTCACTTTTCCACTGTTATTTTTCCATTCTCAACCCCATACTCCTAATGCATTGCAATTAGCCTTACAGGCTCAATCTCACGCTCACTCTTCCACTGTCATTTTTACTTTTACTCGCATTCTCAGTCTCATACTCACTCGCAGTCTCATTCTGGCTCTCATTCTCACTCTTGCTAATACTTTTAC-3’

**3) The peak sequence of H3K18la at the S6K2 promoter site**

5’-ATATATAAGTATGTAATGTACATATTGATGCGCTTACTCATATATTTCTATATTGATACATATATTTGTTTATATATTTATTAATGTTATTATAGGTATTGACATTATGTAATTATTGGTATATATATATTTTTTTTTATGTAATATGCATGTATACATCTATTTGTATGTATGCATTTATTCATAGTTTAGATACAAAGTAATTAAAGATACTGAATATGTTTTTCCCTTGAATTATATAACTGACGCAGATTTGTTGTAATTACACTCCTTATCCTGCTGCAATGCATTGTCATTTAATGTTGAGTAATGGAATCAGGCATTAGTGTAGTAGACTATATAGTATTTATTTTATTATCTTTAGAAGTAATAGATTTTATCAGAGATATAACCAGGGTTTTATTTTAGTTATGATTGCTTGTTAGGGATTTGATATCTTGAGAGATTAAGATAAGAGAATCAGGATTTCCTTTTTTTGAGTACATTACACTTGGTATAAGAATGCATAAGTGAATCACATTTGTGTGTGTGTGTGT-3’

**Supplementary Tables**

**Table S1. The gene identity (ID) used in this study.**

| **Gene** | **Gene ID** |
| --- | --- |
| ATP-dependent translocase (ABCB1) | LOC122243907 |
| phospholipase D1 (PLD1) | LOC122265968 |
| ribosomal protein S6 kinase 2 (S6K2) | LOC122247181 |
| TGF-beta receptor type-1 (TGRB1) | LOC122254409 |
| L-lactate dehydrogenase (LDH) | LOC122260820 |
| histone deacetylase 3 (HDAC3) | LOC122245885 |
| histone deacetylase 1 (HDAC1) | LOC122257912 |
| CREB-binding protein (p300) | LOC122243702 |
| hexokinase (HK) | LOC122247133 |
| atypical protein kinase C (aPKC) | LOC122256299 |
| hypoxia-inducible factor 1-alpha (HIF-1α) | LOC122250593 |

**Table S2. Primers for quantitative real-time PCR.**

| **Primers** | **Sequences (5’-3’)** |
| --- | --- |
| HK Forward | TGACAGAGACGAGAACAC |
| HK Reverse | AATCACTTCACGCTGACT |
| LDH Forward | TGATAAGGAAGAGACTGTT |
| LDH Reverse | CTCATACCAATAAGCCATAA |
| p300 Forward | ATCTGCGTTGCTTACCTA |
| p300 Reverse | TTGGTGTTCTTCTTCTTGTG |
| HDAC1 Forward | TCTTGAACTATGGACTCT |
| HDAC1 Reverse | ATGTTATCAGGACGAATG |
| HDAC3 Forward | TCCGAAGAATACATAGAC |
| HDAC3 Reverse | GAGCAGAAGTCATATAGG |
| S6K2 Forward | AATGCTCACCAGTTGTTC |
| S6K2 Reverse | TTGGTGTTCTTCTTCTTGTG |
| β-actin Forward | CGAGCACGGCATCGTTACTA |
| β-actin Reverse | TTGTAGAAAGTGTGATGCCAGATCT |
| PLD1 Forward | ACCATTAGCAGTTGATAA |
| PLD1 Reverse | CATTCTTCTTCTCCTCTT |
| TGFBR1 Forward | CTTGGTCTGGTCTATTGG |
| TGFBR1 Reverse | GTATGGTAACTGGTATTCCT |
| aPKC Forward | GCTGATGGTAGAACTGAA |
| aPKC Reverse | CAATCTATGTCCTCGTCAT |
| ABCB1 Forward | CTCATACAGCGATTCTAT |
| ABCB1 Reverse | TAACCAACCGATATTCAG |
| HIF-1α Forward | ACAACAACAGCAAGTATCG |
| HIF-1α Reverse | ATGTAGTGTCTCCTCTCAAC |
| S6K2-H4K12la-promoter Forward | GATACACAGAAGAGGATATGGA |
| S6K2-H4K12la-promoter Reverse | CGAAGATTGCGTCACTATG |
| S6K2-H3K18la-promoter Forward | ATCAGGCATTAGTGTAGTAG |
| S6K2-H3K18la-promoter Reverse | GCATTCTTATACCAAGTGTAAT |
| WSSV-specific Forward | TTGGTTTCATGCCCGAGATT |
| WSSV-specific Reverse | CCTTGGTCAGCCCCTTGA |
| TaqMan probe | FAM-TGCTGCCGTCTCCAATAMRA |

**Table S3. Primers for vector construction.**

| **Primers** | **Sequences (5’-3’)** |
| --- | --- |
| Pet28-sumo-LDH-Forward | CGGGATCCATGGCCTCTGTTCCCGAAA |
| Pet28-sumo-LDH-Reverse | CCAAGCTTGTTAGAACTGGATTCCAGCCT |
| Pet28-sumo-HIF-1α-Forward | CCAAGCTTGCATGTGCTTAAGGACTAATTT |
| Pet28-sumo-HIF-1α-Reverse | CCGCTCGAGTTTATGGACTCTGATCTAATGCC |
| pGEX-6p-2-S6K2-Forward | CGGGATCCATGCTGACGGAGGTGCTGAG |
| pGEX-6p-2-S6K2-Reverse | CCCTCGAGTTCAGACCTCCGTCGACGAC |

**Table S4. Sequences of siRNAs.**

| **Oligonucleotides** | **Sequences (5’-3’)** |
| --- | --- |
| LDH-siRNA | AGUUUGAUGAUCUCAUAUGCA |
| LDH-siRNA-scrambled | AGUUUGAUGAUCACAUAUGCA |
| p300-siRNA | AGCAUUGUUUGCUUAUGAAGUGA |
| p300-siRNA-scrambled | AGCAUUGUUUGCAUAUGAAGUGA |
| HDAC1-siRNA | GGUUUACAGUCAUGCAAUACU |
| HDAC1-siRNA-scrambled | GGUUUACAGACUUGCAAUACU |
| HDAC3-siRNA | GCUACAUCCAGGUGUUCAAAC |
| HDAC3-siRNA-scrambled | GCUACAUCCUGGAGUUCAAAC |
| S6K2-siRNA | GCUGUGUUGCUGACUCUAACC |
| S6K2-siRNA-scrambled | GCUGUGUAUCUGACUCUAACC |

**Supplementary figures**

**Fig S1. Influence of virus infection on host glycolysis and internalization.** (A) Effects of virus infection on shrimp glycolysis. Shrimp were infected with WSSV. PBS was included in the injection as a control. At different time after injection, the content of lactate in the hemocytes and intestinal tissues of shrimp was examined (**, *p*<0.01). (B) Impact of WSSV infection on the expression levels of hexokinase and lactate dehydrogenase in shrimp. Shrimp were injected with WSSV or PBS. PBS was used as a control. At different time after injection, the expressions of hexokinase and lactate dehydrogenase in the hemocytes or intestinal tissues of shrimp were examined by quantitative real-time PCR (**, *p*<0.01). (C) Detection of the activity of hexokinase or lactate dehydrogenase in the hemocytes or intestinal tissues of WSSV-infected shrimp. PBS was included in the injection as a control (**, *p*<0.01). (D) Impact of virus infection on internalization. Shrimp were injected with WSSV or PBS. PBS was included in the injection as a control. At different time after injection, the phagocytic percentage of shrimp hemocytes was examined (*, *p*<0.05) (left). The representative phagocytosed virions in hemocytes were indicated on the right. Scale bars, 40 μm.

**Fig S2. Sequence alignment of catalytic domain of HDAC1, HDAC3 and p300 proteins of shrimp and human being.** The amino acid sequences of the catalytic domains of shrimp and human *p300*, *HDAC1* and *HDAC3* were obtained in the NCBI database (<http://www.ncbi.nlm.nih.gov/>). The pair-wise sequence alignments were performed using DNAMAN software.

**Fig S3. Kyoto encyclopedia of genes and genomes (KEGG) analysis of the 75 upregulated target genes in the WSSV-infected shrimp with H3K18la and H4K12la.**

Fig S1

A


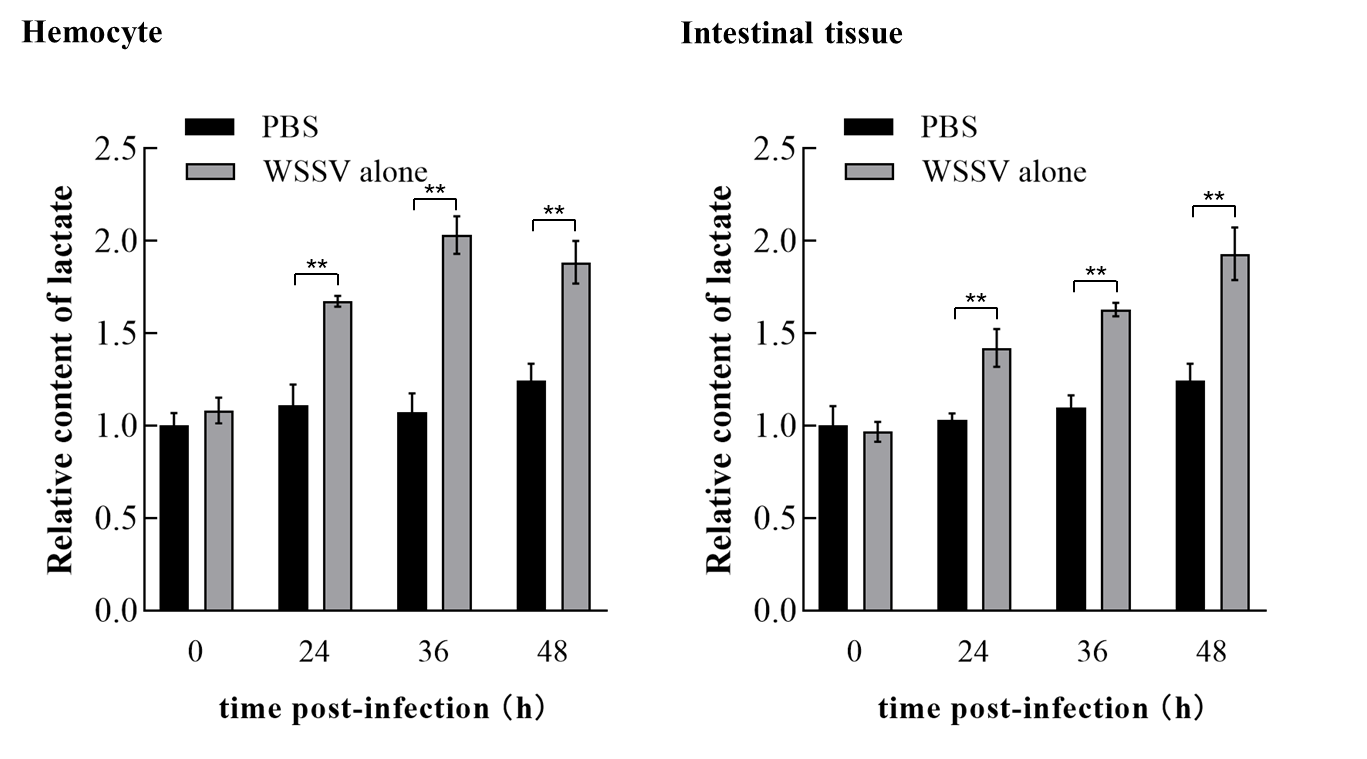


B


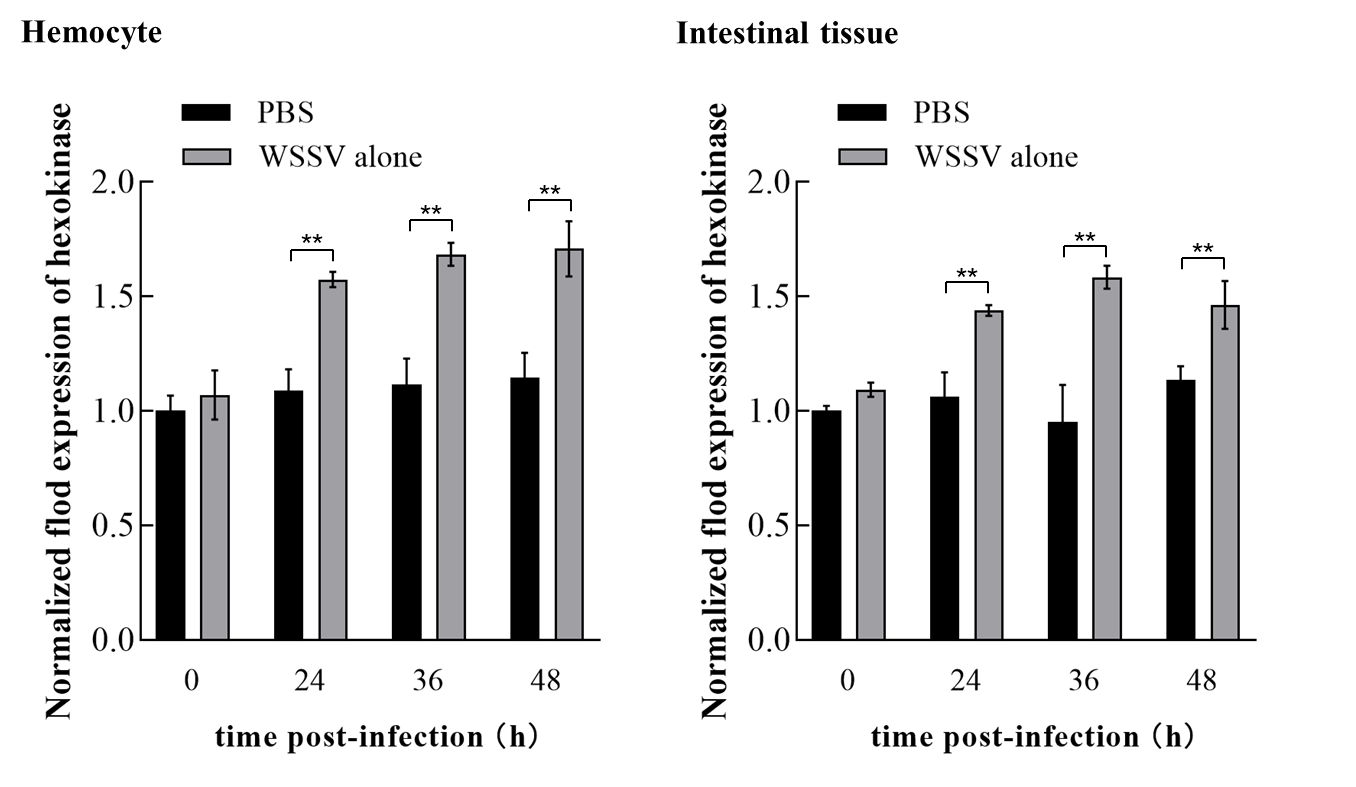


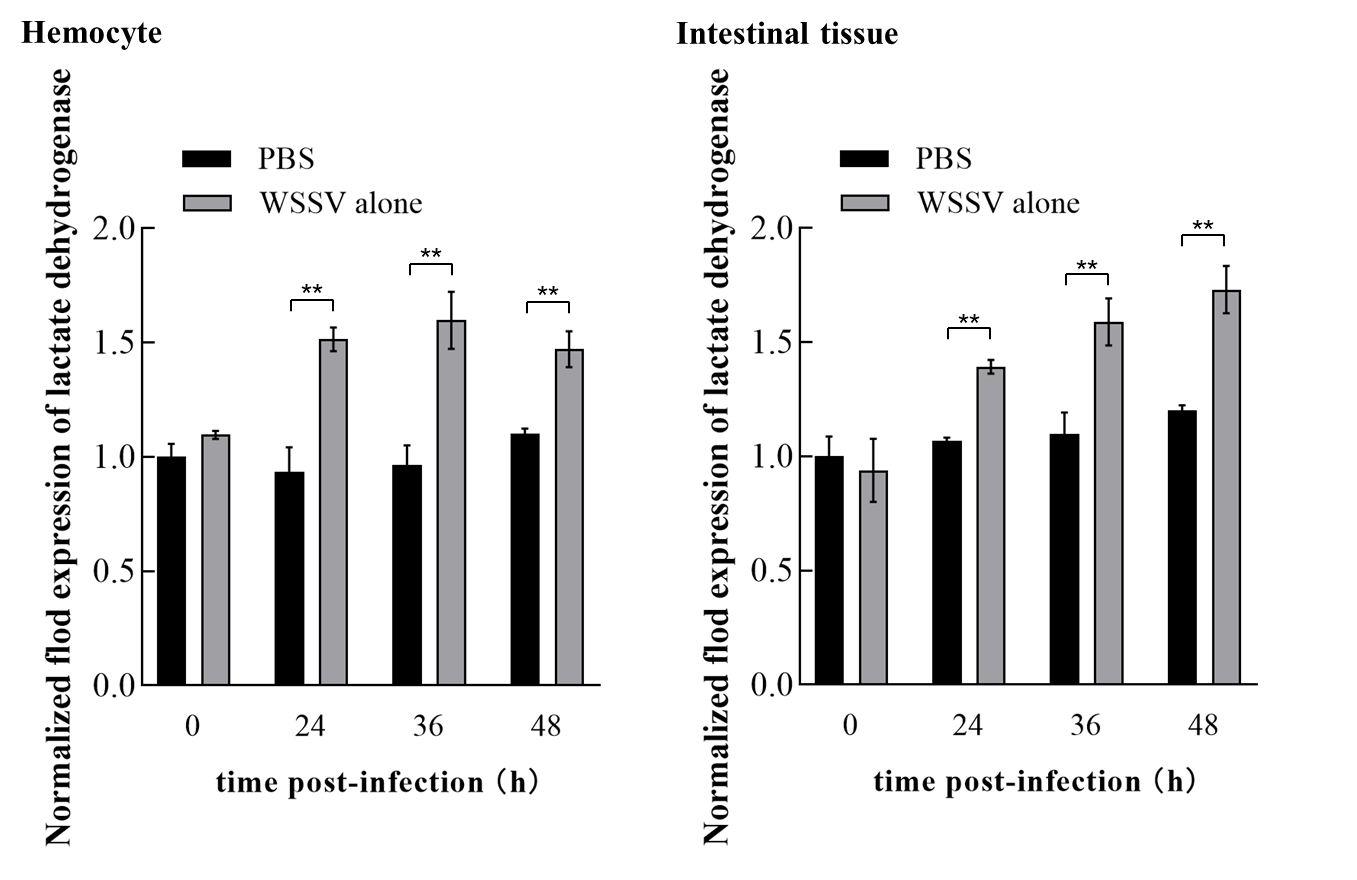


C


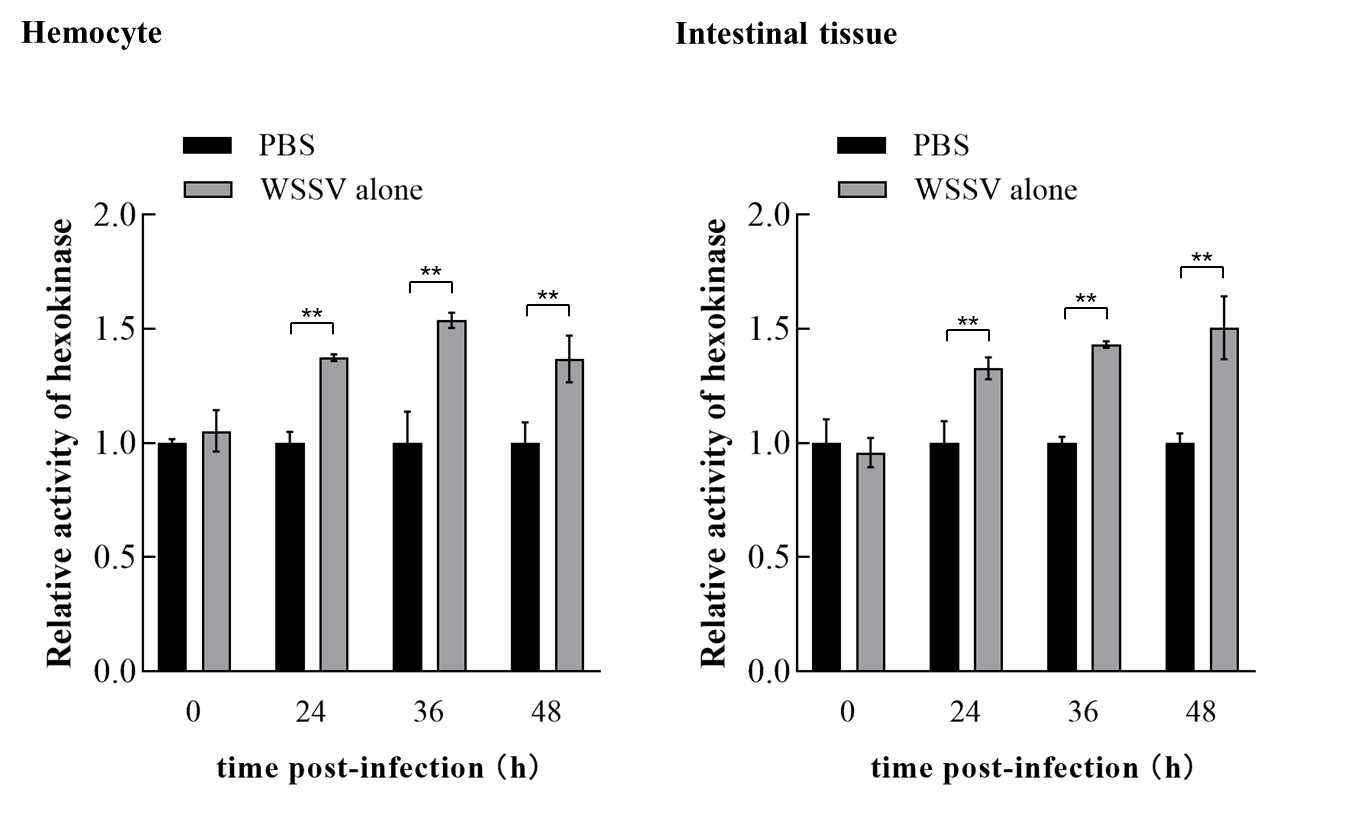


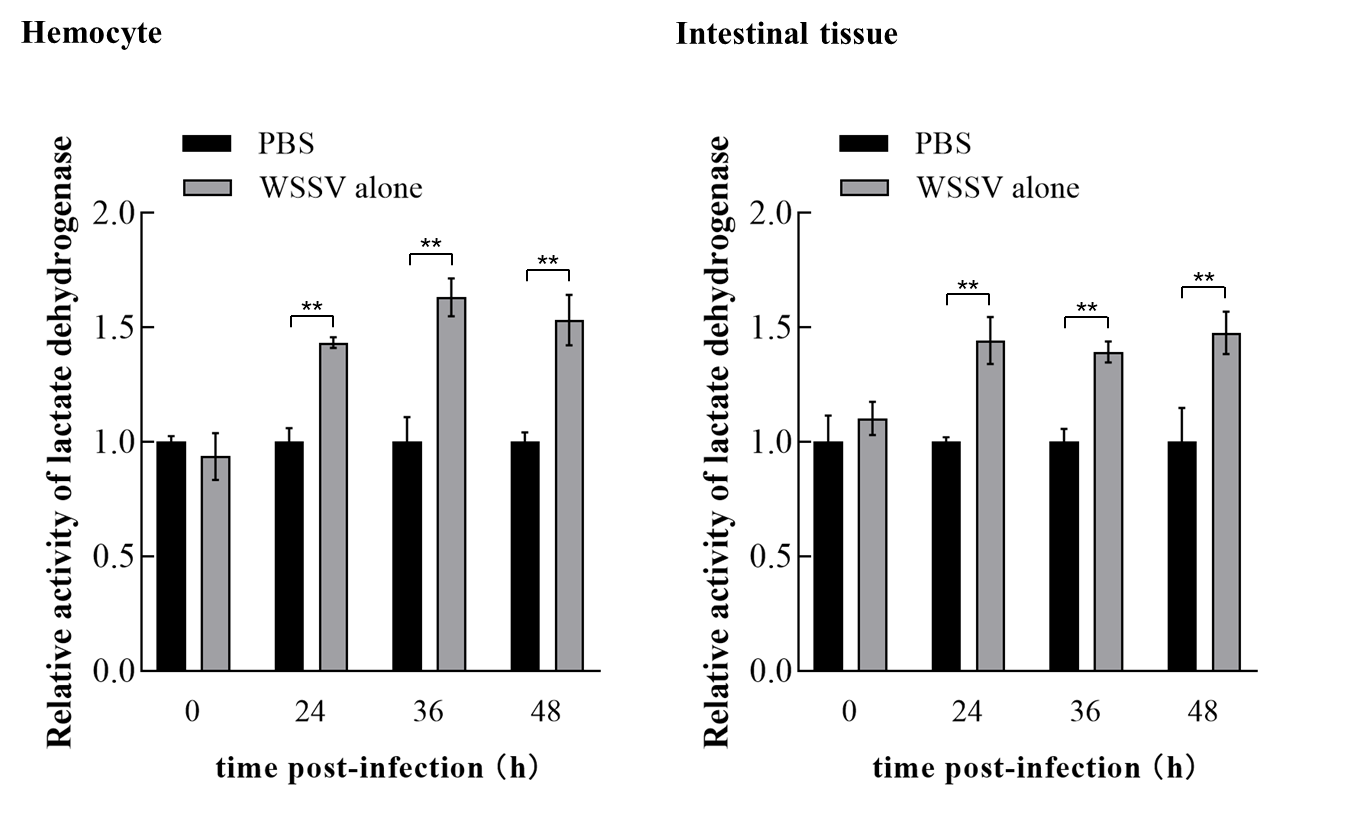


D


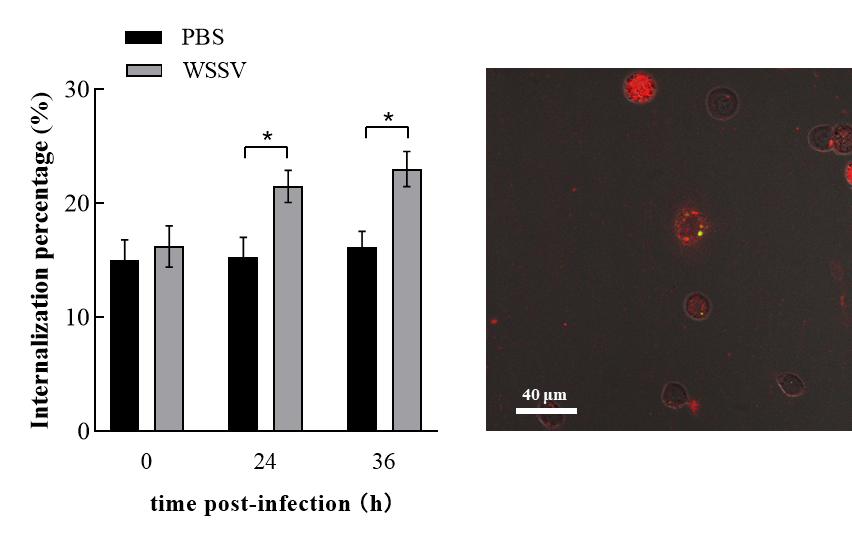


Fig S2


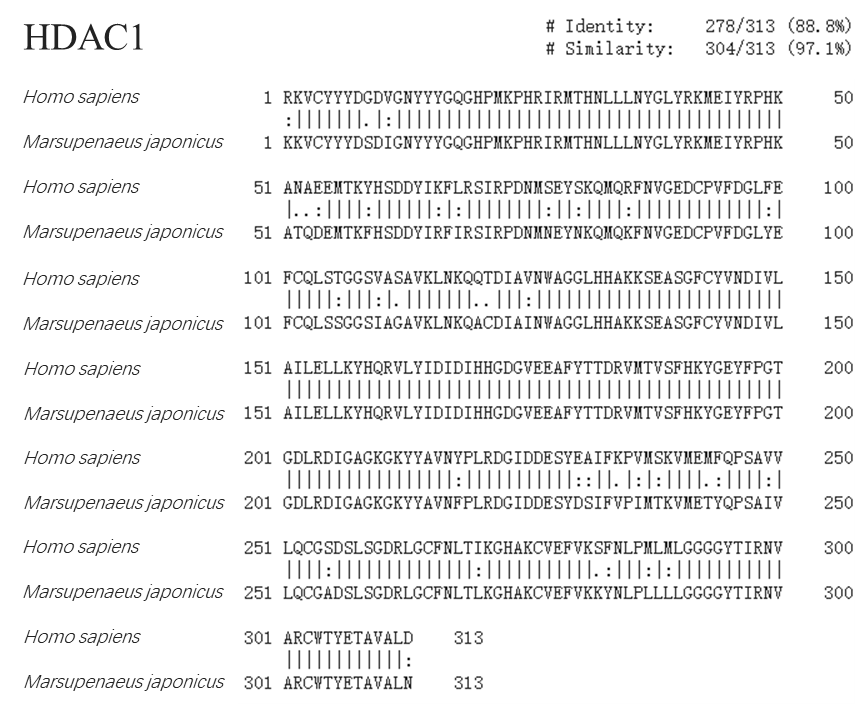


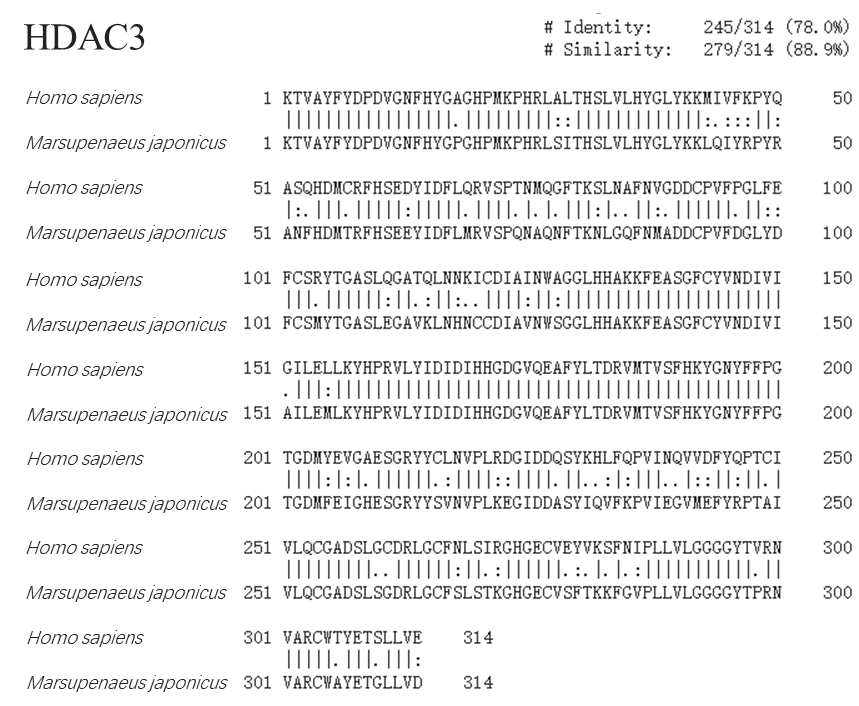


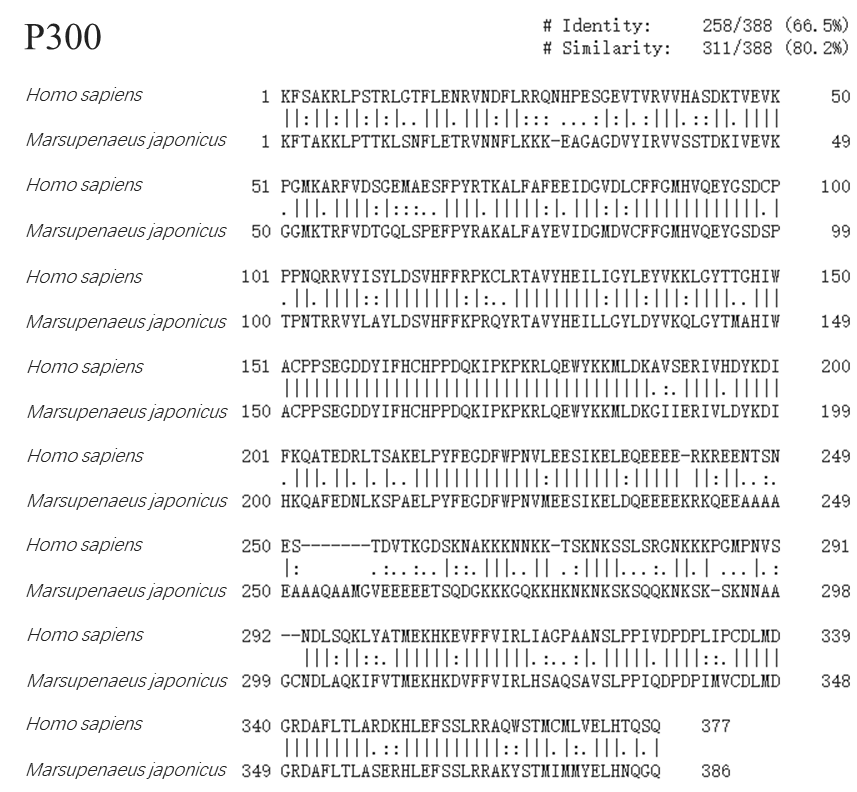


Fig S3


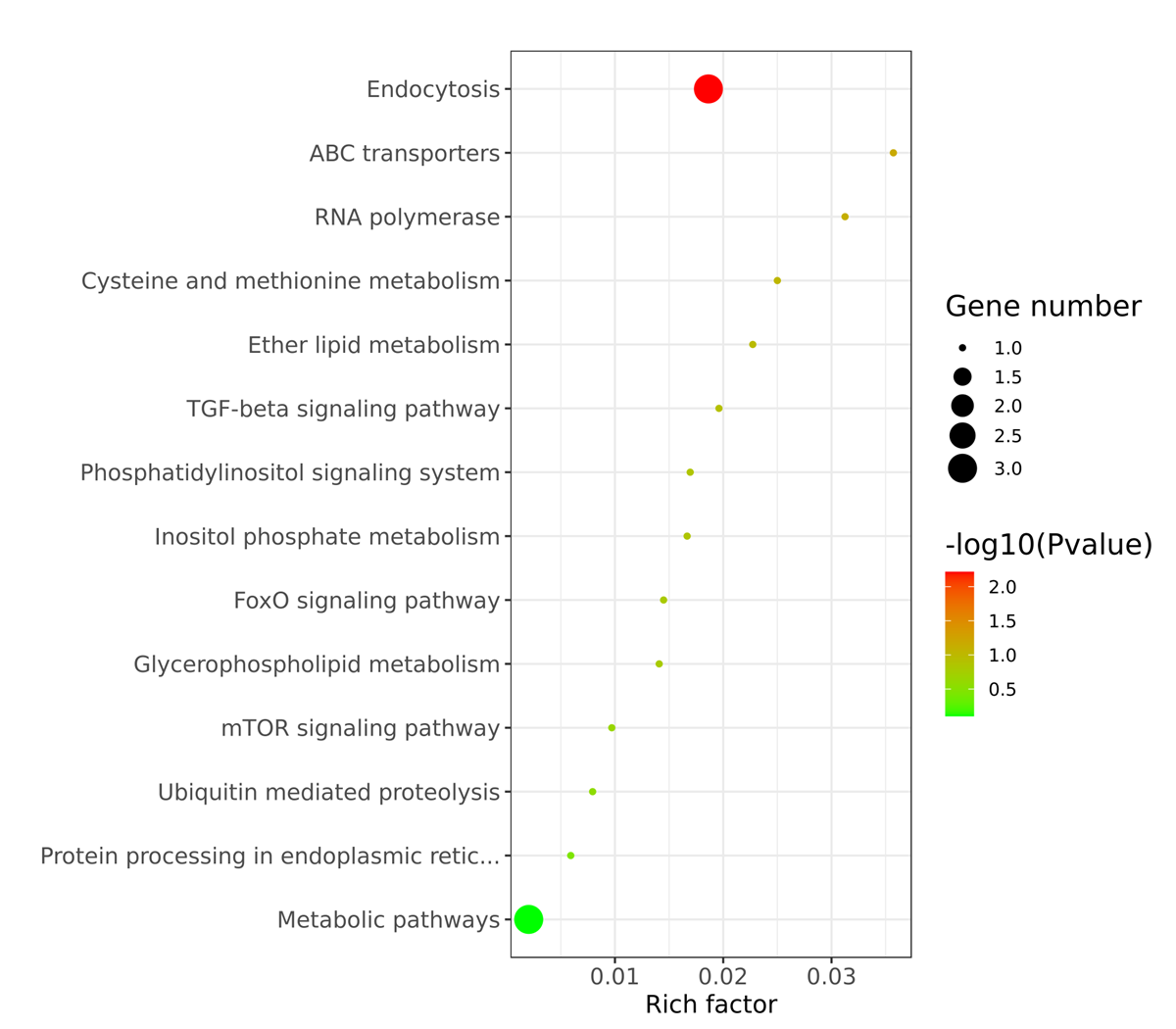

Supplement: Supplementary file 1 — Supporting Information [file ADVS-11-2401017-s001.docx]
